# The complementary-competitive interplay of consumer information search and experience sharing: Implications for targeted advertising and firm performance

**DOI:** 10.1371/journal.pone.0340849

**Published:** 2026-10-28

**Authors:** Wu Dan, Zhao Jiang

**Affiliations:** 1 Cai Yuan-pei School of Art and Design, Shaoxing University, Shaoxing, China; 2 Ma Yin-chu School of Economics, Management & Laws, Shaoxing University, Shaoxing, China; 3 Shaoxing Institute of Yue Business Research, Shaoxing, China; University of Naples Federico II: Universita degli Studi di Napoli Federico II, ITALY

## Abstract

The proliferation of targeted media platforms enables firms to deliver tailored advertising to specific consumer segments, yet the heterogeneous influences of pre-purchase information search and post-purchase sharing on targeted advertising performance remain underexplored in extant research. This paper constructs a two-period symmetric duopoly game model with fully rational firms and consumers to analyze theoretical associations between two distinct consumer information behaviors and firms’ equilibrium advertising and profit outcomes. We set homogeneous products, symmetric competitors, and two types of consumers (loyal buyers and comparison shoppers) to explore the strategic interplay of corporate advertising, pricing and consumer information activities. This model adopts a differentiated search cost framework: digital information access incurs zero monetary expenses, yet endogenous cognitive information overload creates non-negligible mental frictions. Search intensity s is the consumer’s generic choice variable; $s^{*}$ denotes the endogenous equilibrium search intensity solved from consumer utility maximization (not an exogenous primitive parameter), which lies within interior range (0,1) rather than corner solution s=1. Experience‑sharing intensity e is treated as an exogenous symmetric parameter for analytical tractability. Model-derived predictions are summarized as follows: (1) Consumer information search acts as a complementary force for targeted ads by lowering information frictions, lifting conversion rates and raising equilibrium profitability within valid parameter ranges; (2)Experience sharing generates dual competitive effects: moderate peer reviews expand market awareness, while excessive price-related sharing intensifies cross-brand price rivalry and suppress firm returns; (3) The joint interaction of search and sharing shapes equilibrium advertising intensity: optimal ad spending falls as primitive cognitive‑friction parameters shift to raise endogenous equilibrium search intensity $s^{*}$, and firms need context-dependent strategies to cope with consumer sharing. Theoretically, this work unifies pre-purchase search and post-purchase sharing into a single dynamic duopoly framework to fill research gaps that separate the two consumer behaviors. Practically, the model provides neutral theoretical insights; tentative suggestions for advertising‑message content represent directions for future empirical testing rather than formal model outputs. All theoretical conclusions derived in this paper hold only within the feasible interior-equilibrium parameter domain defined in Text S2.9. Numerical simulation figures serve purely as numerically illustrated examples; formal mathematical proofs are provided in the appendices. All conclusions are mathematical predictions derived under preset model assumptions, rather than empirically validated causal relationships.

## 1. Introduction

Advertising is a pivotal strategic decision in marketing, and the advancement of data mining and big data analytics has revolutionized targeted advertising, enabling firms to deliver tailored messages to specific consumer segments and reduce advertising waste [1]. According to MAGNA’s 2023 report, global digital advertising spending reached $683 billion, accounting for 64% of total advertising expenditure, with targeted advertising driving most of this growth. Despite its prevalence, targeted advertising effectiveness is increasingly shaped by consumer proactive information behaviors—consumers now actively search for product information before purchase and share consumption experiences with peers after purchase [2,3]. These behaviors may either complement or undermine targeted advertising, yet their heterogeneous impacts and interactive effects remain underexplored in existing literature.

Prior research on targeted advertising has focused on firm strategies (e.g., pricing, advertising intensity) or single consumer behaviors (e.g., search costs, advertising avoidance) [4–6]. For instance, Ursu et al. explored the relationship between targeted advertising and costly consumer search, but assumed search costs are positive [6], which sits in tension with digital‑market environments characterized by zero direct monetary access price, with positive cognitive or effort cost [7]. Meanwhile, studies on experience sharing have primarily focused on its positive impact on brand loyalty [8], ignoring its potential role in facilitating inter-firm price comparison and consumer switching. Few existing studies have integrated information search (pre-purchase, yielding endogenous $s^{*}$) and experience sharing (post-purchase, exogenous parameter e) into a unified duopoly game framework to examine their interplay on targeted advertising effectiveness and firm performance. This claim is conditional on existing searches of relevant literature; we do not assert absolute universal nonexistence of prior work. This research gap limits our understanding of how firms should optimize targeted advertising strategies in the era of proactive consumer information behaviors.

This study aims to fill this gap by addressing three core research questions (RQs):

**RQ1:** Within a duopoly market, what conditional theoretical relationship does the model predict under its stated assumptions between pre‑purchase consumer information search and firms’ targeted‑advertising intensity and equilibrium profits?
**RQ2:** How does consumer experience sharing (post-purchase, exogenous parameter e) influence duopoly competition, firms’ pricing strategies, and long-run performance?
**RQ3:** What is the interactive theoretical association between information search and experience sharing, and what context-dependent advertising-pricing logics can be derived strictly from model equilibrium outcomes for firms?

To answer these questions, we employ a two-period duopoly game-theoretic model with rational consumers and firms, assuming homogeneous products, symmetric firms, and two consumer segments (loyal and comparison consumers). A complete, authoritative game timeline defining players, actions, information sets, timing, observations, and payoff realization for each period is provided in Section 3.1. The model captures inter‑temporal spillover: Period‑1 post‑purchase experience sharing affects Period‑2 pre‑purchase consumer information conditions.This study makes three key contributions. First, theoretically, it enriches the targeted advertising literature by integrating two distinct proactive information behaviors of consumers into a unified analytical framework, revealing their complementary-competitive linkage. Second, methodologically, it extends prior duopoly models by adopting zero direct monetary access price, with positive cognitive or effort cost, while carefully distinguishing generic choice variable s, endogenous equilibrium $s^{*}$, and primitive cognitive‑friction parameters. Third, practically, it provides conditional theoretical outputs for firm strategy; we explicitly separate formal mathematical results from speculative directions awaiting future empirical work.

## 2. Literature review

### 2.1. Targeted advertising and duopoly competition

Targeted advertising has emerged as a critical strategy for firms to mitigate information asymmetry and improve advertising efficiency [9]. Early studies focused on the superiority of targeted advertising over mass advertising, showing that tailored messages can increase consumer purchase intention by matching individual preferences [4,10]. In duopoly settings, Ben Elhadj-Ben Brahim et al. developed a Hotelling-type model and found that firms target ads to consumers closer to rivals when advertising costs are high [11]. However, these studies assume consumers are passive recipients of advertising, ignoring their proactive information behaviors (e.g., search, experience sharing) that are prevalent in digital markets. Nevertheless, this prior study does not incorporate joint pre-purchase search and post-purchase sharing mechanisms, which constitute important elements within our duopoly model. More recent studies have integrated consumer search into targeted advertising models, but they focus on costly search, which is less relevant for online environments where information is easily accessible [7]. Our analysis differentiates zero‑monetary access price from positive cognitive‑effort frictions and adds inter‑temporal peer‑information spillovers.

### 2.2. Consumer information search and advertising effectiveness

Consumer information search is a pre-purchase behavior that reduces information asymmetry and influences purchase decisions [12]. Schmidt & Spreng proposed an economic model of information search, highlighting that search willingness is driven by consumer ability, perceived costs, and potential utility gains [13]. This work focuses on static individual search decisions but does not embed search within a two‑period duopoly advertising‑pricing game. In the context of targeted advertising, Ge et al. found that search activity raises consumer sensitivity to advertising relevance [2], while Burguet (2023) demonstrated that targeting ads can stimulate consumer search [7]. Neither paper jointly models search together with post-purchase peer experience sharing spillovers. However, existing literature fails to analyze how search behavior complements targeted advertising to lift corporate profits, nor does it jointly model search with post-purchase experience sharing. Our research addresses this gap by constructing a two-period dynamic framework that unifies the two consumer behaviors.

### 2.3. Consumer experience sharing and market dynamics

Experience sharing is a post-purchase peer communication mechanism covering product quality and pricing information [3]. Some prior papers examine knowledge‑sharing within organizations or forecast‑information exchange within supply‑chain systems [14, 15]. Those studies investigate different institutional contexts and are not direct theoretical foundations for consumer‑level peer experience sharing in duopoly advertising‑pricing competition. Kim & Jang studied social media sharing but did not link this behavior to duopoly advertising competition [16]. Ghose A et al. noted interactions between social information and consumer search but lacked formal game modeling [17]. Recent advances in artificial‑intelligence‑driven marketing tools have further expanded the practical scope of targeted advertising, bringing new opportunities alongside technical and ethical challenges for audience targeting, personalized content generation, and ad performance optimization [18]. Emerging research highlights that modern targeted advertising systems must account for real‑world targeting frictions and heterogeneous consumer perspectives, rather than assuming perfect audience identification [19,20]. Prior theoretical work has examined how targeting precision shapes market competition and industry regulatory outcomes within competitive market settings [21, 22]. Nevertheless, existing game‑theoretic models seldom jointly incorporate pre‑purchase consumer search and post‑purchase peer experience‑sharing behaviors when evaluating targeted‑advertising equilibria [23]. Their analysis provides verbal conceptualization without backward-induction equilibrium solutions for targeted advertising and mixed-strategy pricing. This paper formalizes experience sharing as a dual-edged competitive mechanism to fill this research vacuum.

### 2.4. Research gap synthesis

In summary, three core limitations exist in extant research: (1) Most studies treat consumers as passive information receivers rather than active information seekers and sharers; (2) Prior work only analyzes search or sharing in isolation, without exploring their joint interactive effects; (3) Traditional search models commonly assume positive monetary search costs. This diverges from digital environments featuring zero direct monetary access price, with positive cognitive or effort cost. This paper constructs a two-period duopoly game model integrating zero-monetary-cost pre-purchase search and post-purchase sharing to analyze their combined influences on targeted advertising and firm profitability.

Each identified research gap above traces directly back to the literature reviewed in Section 2.1–2.3. To resolve a critical theoretical ambiguity raised by prior zero-cost search frameworks, this study explicitly distinguishes two separable dimensions of consumer search costs. First, all digital product information browsing carries zero direct monetary or time payment costs, consistent with modern e-commerce and social media market reality. Second, continuous cross-brand information screening generates cognitive fatigue and information overload, captured via a strictly concave search utility function $u_{s}(s)$. Diminishing marginal utility of additional information search creates an endogenous upper bound on search willingness, which guarantees an interior equilibrium search intensity s∈(0,1) and rules out the extreme full-search corner case s=1. This dual-cost behavioral foundation eliminates logical contradictions between free digital information and incomplete consumer information acquisition, and all mathematical derivations for this mechanism are documented in S1 Text, Sections A1 and A8.

## 3. The model

### 3.1. Model overview and key assumptions

**Ethics Statement:** Not applicable. This study consists of theoretical modelling and did not involve human participants, animals, biological materials, or field research.

Unified authoritative game timeline (each period repeats this sequence):

Stage 1: Both firms simultaneously choose pure‑strategy targeted‑advertising intensity $\phi_{i}$. Advertising cost $A(\phi_{i})$ is incurred within the current period.

Stage 2: Conditional on realized advertising profile, firms play mixed‑strategy pricing subgame for comparison‑shopper segment; loyal consumers buy at price not exceeding reservation value v.

Consumers make purchase decisions; Period‑1 purchasers generate peer‑experience information spillover governed by exogenous scalar e, which shapes Period‑2 consumer utility and demand.

In this setup, we assume that both consumers and firms act rationally, maximizing their utility and profits, respectively. While there may be imperfect information about product matching value, we assume complete knowledge of market rules and strategic options for all participants. We develop a two-period duopoly game model (t = 1,2) with two symmetric firms (Firm A and Firm B) producing homogeneous products. The model focuses on strategic interaction between firms’ targeted advertising/pricing decisions and two consumer behaviors: pre-purchase information search and post-purchase experience sharing. Both firms produce homogeneous products with zero constant production cost for analytical simplicity. Each firm owns an identical base group of loyal consumers. The loyal consumer share for each firm is defined as α, meaning loyal buyers will only purchase from their home brand as long as the price does not exceed the consumer’s reservation value v. In contrast, a proportion β of consumers are comparison shoppers who switch brands after cross-price comparison. The total market mass is normalized to 1, satisfying 2α+β=1, or equivalently α=(1−β)/2. The competitive advertising interaction between the two firms is illustrated in Fig 1. A systematic mapping of core model assumptions, mathematical implementations, derived propositions, and supporting figures is provided in Table 1. In this model, Firms simultaneously adopt pure‑strategy targeted advertising intensity in the first stage. Conditional on predetermined advertising configurations, subsequent price competition occurs. Specifically, loyal consumers face deterministic pricing constrained by product reservation value, while the contestable comparison‑shopper segment generates a mixed‑strategy pricing equilibrium. This sequential separation clarifies the intrinsic linkage between advertising strategic decisions and pricing equilibrium outcomes.

**Fig 1:**
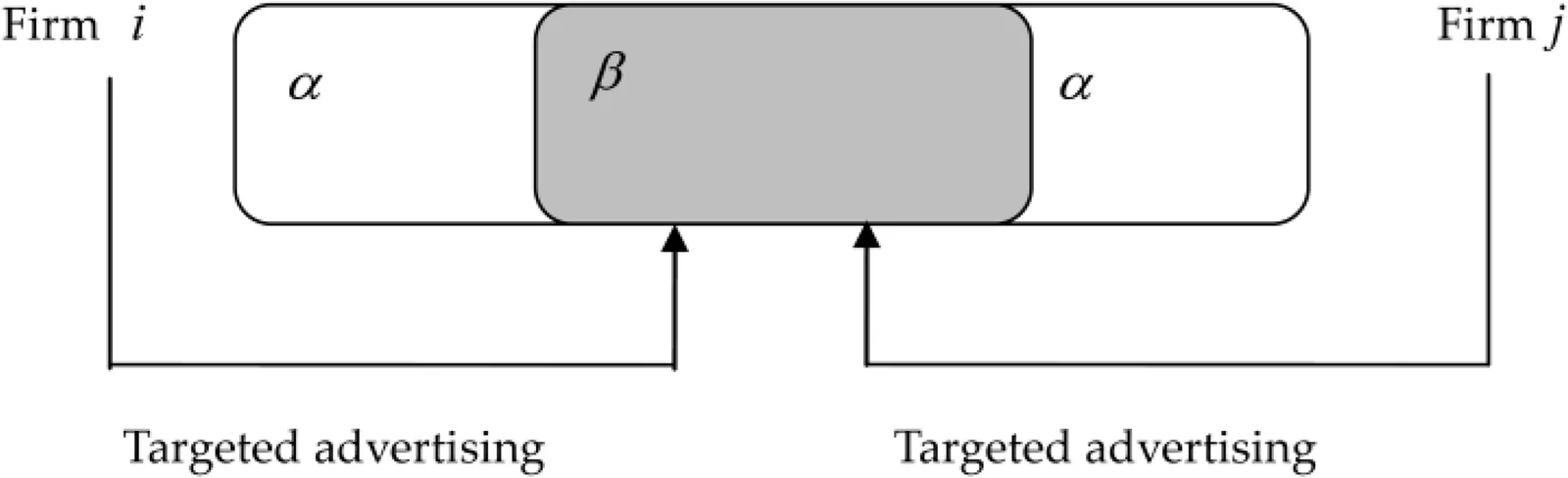
Duopolistic firms compete in the homogeneous market by delivering internet-based targeted advertising.

**Table 1:** Mapping of core modelling assumptions to the seven formal theoretical propositions.

No.	Core Modelling Assumption	Mathematical Implementation (Equation / Appendix)	Corresponding Propositions	Supporting Figures
A1	Symmetric duopoly with two identical firms producing homogeneous products at zero constant marginal cost	Section 3.1; demand functions in S1 Text.3–A.4; profit function Eq. (1)	Prop. 1(a)–(c), Prop. 2	Fig 1, Fig 2
A2	Two consumer segments: loyal buyers (share α) and comparison shoppers (share β), with 2α+β=1	Section 3.1; mutually-exhaustive demand decomposition on S1 Text.3; Eq. (1) demand terms	Prop. 2, Prop. 5, Prop. 7	Fig 1, Fig 4
A3	Zero monetary search cost for digital information access; positive cognitive friction from information overload	Section 3.3; concave search utility $u_{s}(s)$ in S1 Text.1; dual-cost derivation S1 Text.8	Prop. 3, Prop. 4	Fig 2, Fig 3
A4	Endogenous equilibrium search intensity $s^{*}\in (\text{0},\text{1})$ derived from consumer utility maximisation (not exogenously imposed); s = generic consumer choice variable	Section 3.2–3.3; FOC- SOC in S1 Text.1; boundary check S1 Text.8; interiority bounds S2 Text.9	Prop. 1(c), Prop. 3, Prop. 4, Prop. 7	Fig 2, Fig 3, S1 Fig
A5	Peer experience sharing as inter-temporal information spillover: Period-1 sharing enters Period-2 consumer utility; consumers gain no direct utility from producing their own reviews	Section 3.2; utility function $U=v-p+U_{search}(s)+U_{share}(e)$; S1 Text.2	Prop. 5, Prop. 7	Fig 4, Fig 5
A6	Aggregate sharing intensity e: exogenous symmetric scalar for analytical tractability; limitations noted Section 8.4; e explicitly enters Period‑2 utility, demand and profit structure in S1 Text .4	Section 3.1; spillover term in Period-2 demand Eq. (1); limitation noted Section 8.4	Prop. 5, Prop. 7,	Fig 4, Fig 5
A7	Convex advertising cost A(φ) = λφ²/2 with λ > 0	Section 3.4; cost term in profit function Eq. (1); FOC derivation S2 Text.1	Prop. 1(b), Prop. 2, Prop. 3	Fig 2 S2 Fig
A8	Two-period discounted total profit $\Pi_{i}=\Pi_{i}^{\text{1}}+\delta \Pi_{i}^{\text{2}}$ with discount factor δ ∈ (0,1]. Advertising cost incurred in each period	Section 3.1; Eq. (1); backward induction S2 Text.1	Prop. 1, Prop. 4,	Fig 2, Fig 3, S3 Fig
A9	Pure-strategy advertising intensity $\varphi_{i}$ chosen simultaneously; mixed-strategy pricing equilibrium for comparison-shopper segment (modified Varian-type sales model)	Section 3.1; expected conditional price $E[p_{i}^{t}(\phi^{*})]$ in Eq. (1); full mixed‑strategy derivation S2 Text.4,	Prop. 1(a), Prop. 2, Prop. 5	Fig 1, Fig 4
A10	Fully rational firms and consumers; complete knowledge of market rules and strategic options	Section 3.1; utility maximization S1 Text.1; profit maximization S2 Text.1–B.3	All formal Propositions 1‑7	All figures

Note. Each behavioral or structural assumption is linked to its mathematical realization, the propositions that build upon it, and the figures that provide numerical illustration. All formal propositions apply only within the feasible interior‑equilibrium parameter domain defined in S2 Text.9.

**Fig 2:**
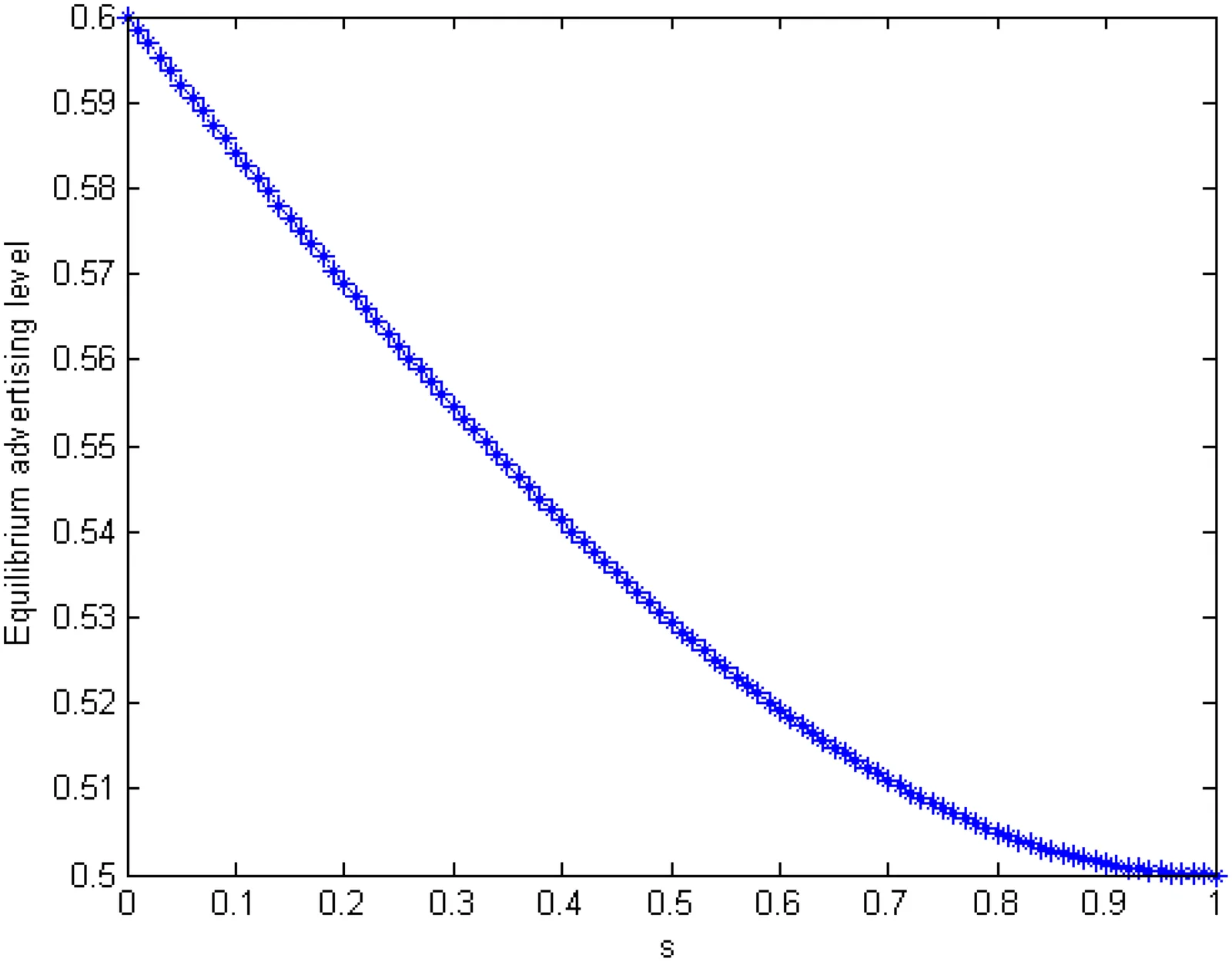
Numerical plot of equilibrium targeted advertising intensity against consumer search intensity. Note: The horizontal‑axis labelled s plots endogenous equilibrium search intensity $s^{*}$. Variation in $s^{*}$ is generated by perturbing primitive cognitive‑friction parameters; $s^{*}$ is not treated as an exogenous input variable. Baseline parameters α=0.3, β=0.4, λ=0.8, δ=0.9. This figure serves purely as numerically illustrated example; formal mathematical proofs are provided in S2 Text.

**Fig 3:**
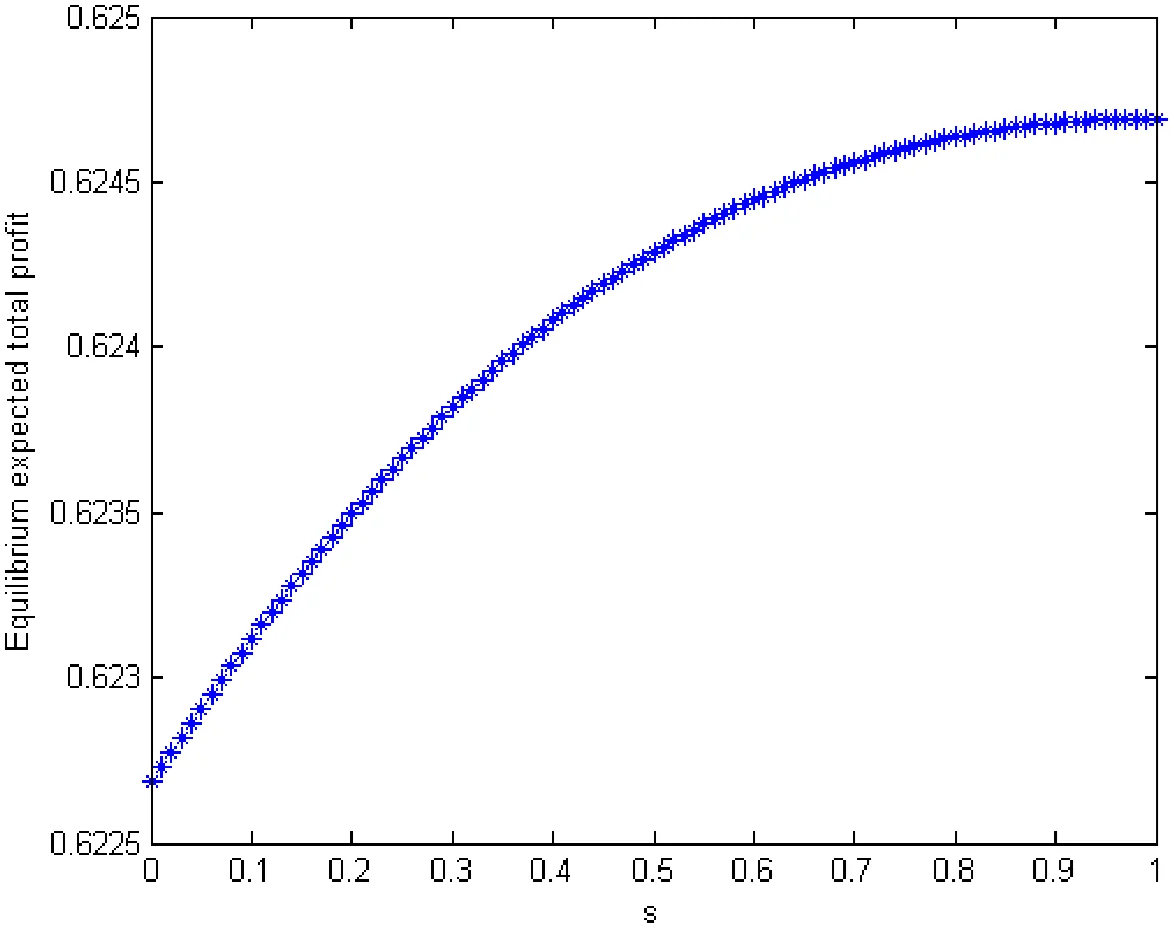
Two-period total equilibrium profit as a function of endogenous equilibrium search intensity $s^{*}$. Note: Horizontal‑axis plots endogenous equilibrium search intensity $s^{*}$, variation generated by perturbing primitive cognitive‑friction parameters. Baseline parameters α=0.3, β=0.4, λ=0.8, δ=0.9. This figure serves purely as a numerically illustrated example; formal mathematical proofs are provided in S2 Text.

**Fig 4:**
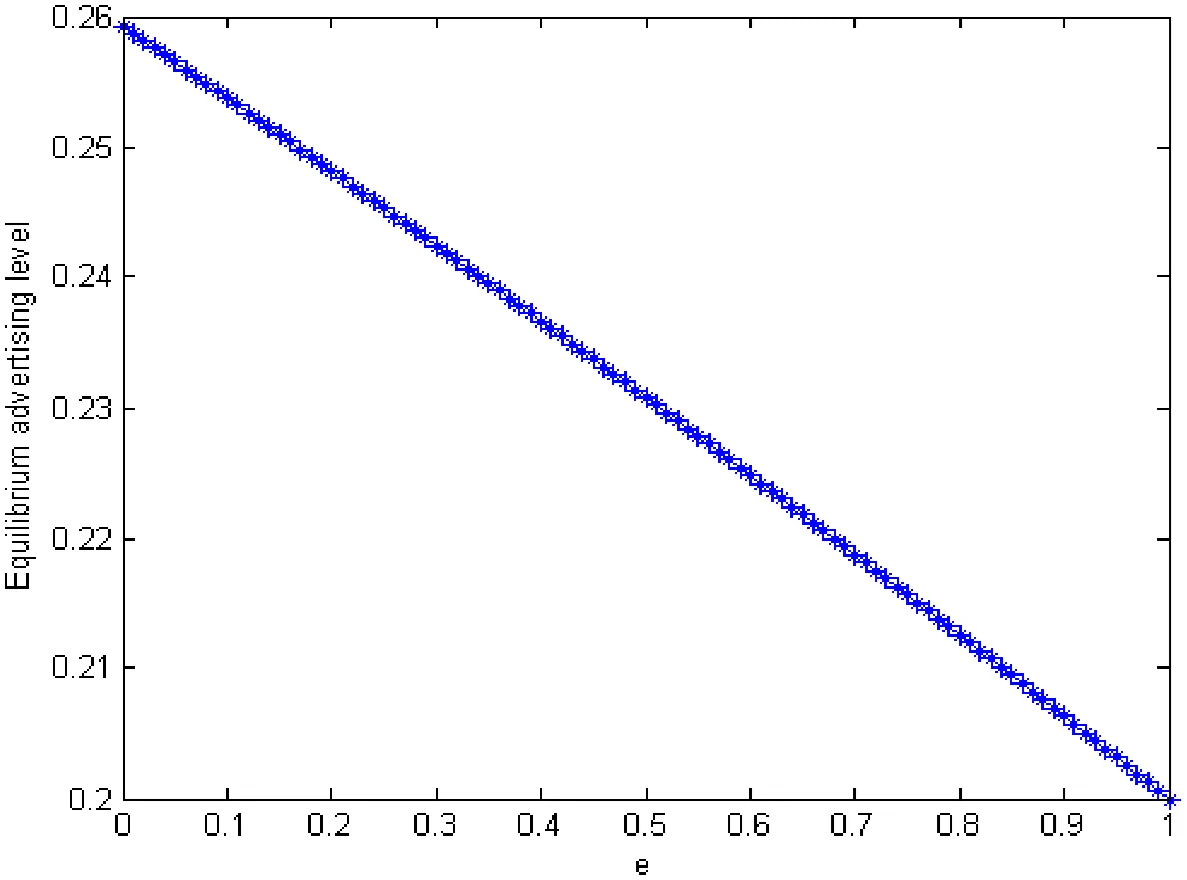
Variation of symmetric equilibrium advertising intensity with exogenous experience sharing intensity parameter e. Note: e is an exogenous symmetric spillover parameter. Baseline parameters α=0.3, β=0.4, λ=0.8, δ=0.9. This figure serves purely as a numerically illustrated example; formal mathematical proofs are provided in S2 Text.

**Fig 5:**
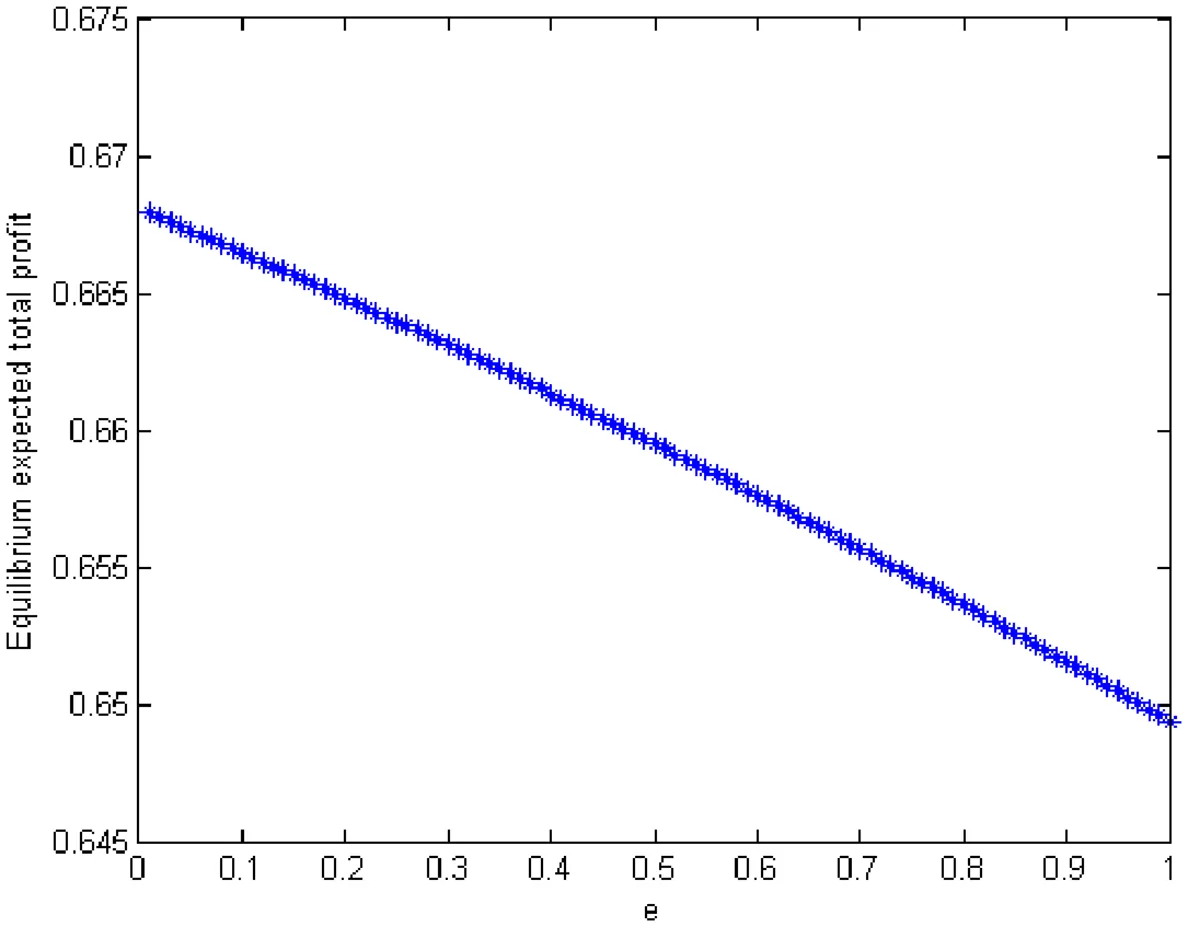
Inverted-U relationship between two-period equilibrium profit and experience sharing intensity e. Note: e is an exogenous symmetric parameter. Baseline parameters α=0.3, β=0.4, λ=0.8, δ=0.9. This figure serves purely as a numerically illustrated example; formal mathematical proofs are provided in S2 Text.

We assume zero-cost information search, which reflects reality of digital platforms where consumers access product information easily [7]. In contrast to prior literature assuming positive monetary search costs, this study distinguishes two layers of search cost: zero direct monetary cost for digital information access, and endogenous cognitive friction arising from information overload. Even with free information, consumers face diminishing marginal utility from continuous browsing, which endogenously restricts search intensity s to an interior range (0,1) rather than the corner solution s=1. This assumption resolves the theoretical paradox raised by passive zero-cost search settings. Search intensity s∈[0,1] refers to the proportion of cross‑brand product information that consumers actively process (generic choice variable); $s^{*}$ is the utility‑maximizing equilibrium outcome. Specifically, s=0 means consumers obtain no information beyond brand advertising, while s=1 represents full acquisition of all market information. The concave cognitive friction ensures that consumers always choose an endogenous optimal search intensity ${\text{s}}^{*}\in (\text{0},\text{1})$ in equilibrium. Rational buyers will voluntarily choose an interior optimal search intensity to balance information benefits and mental fatigue, which logically explains why s never equals 1 in equilibrium. This dual-cost distinction (zero monetary cost + positive cognitive cost) eliminates theoretical contradictions in the model’s behavioral foundation.

Consumers can obtain product information through three channels: brand-targeted ads, voluntary pre-purchase search, and peer post-purchase experience sharing. We distinguish experience sharing from pure price-comparison websites: price platforms only list numerical prices, while consumer sharing transmits both price signals and subjective quality evaluations via social media and word-of-mouth. We set sharing intensity e as an exogenous symmetric scalar parameter to retain analytical tractability given the complexity of the two‑period duopoly framework with endogenous search and mixed‑strategy pricing. The baseline model contains only a scalar exogenous sharing‑intensity parameter e. It does not formally model endogenous content‑composition shifts between quality‑focused versus price‑focused user‑generated content. We explicitly acknowledge this stylized simplification and propose endogenizing sharing intensity as a viable direction for future extension in this research limitations section.

Each firm sets advertising intensity ϕ∈[0,1] and single-period price $p_{i}^{t}$. Superscript t denotes the time period, and the total discounted two-period profit of Firm i is defined as $\pi_{i}=\pi_{i}^{\text{1}}+\delta \pi_{i}^{\text{2}}$, where δ∈(0,1] represents the intertemporal discount factor.

### 3.2. Supplementary consumer utility foundation

To solidify the model’s behavioral logic, we explicitly define the consumer utility function as follows:$\;U=v-p+U_{search}(s)+U_{share}(e)$, where v  stands for reservation value, p is the product price, $U_{search}$ captures utility gains from self-initiated product inquiry, and $U_{share}$ denotes information spillover value from peer experience exchange. We further clarify the economic connotation of each utility component to eliminate conceptual ambiguity: $U_{search}(s$represents the utility improvement generated by consumers’ active pre-purchase information screening behavior. Critically,${\;U}_{share}(e)$ exclusively captures the information-processing utility obtained by reading other consumers’ post-purchase sharing content, rather than utility derived from producing one’s own reviews. Peer experience sharing acts as a purely inter‑temporal information spillover: consumers who complete purchases and generate sharing content in Period 1 receive no direct utility compensation, while consumers making purchase decisions in Period 2 benefit from this aggregated peer information pool. Search intensity s moderates the efficiency of consumers utilizing existing peer sharing information, rather than directly determining the volume of market sharing behavior. Consumers completing purchases in Period 1 generate peer‑information spillovers at aggregate intensity e. These spillovers enter the utility function of consumers making purchase decisions in Period 2.

All demand formulas in Section 3.2 are fully derived from utility maximization, with step-by-step algebraic calculations attached in S1 Text.2‑A.4. S1 Text .3 provides mutually‑exclusive, exhaustive demand decomposition for each period, algebraic demonstration of unit‑market‑mass conservation, and tie‑breaking allocation rules for mixed‑strategy outcomes. Loyal consumers remain fully captive up to reservation value v**.**

### 3.3. Dual search cost mechanism

We formalize the critical search cost assumption to resolve the conceptual tension between zero-cost digital access and incomplete consumer search intensity s<1.

Zero monetary search cost: Consumers face no explicit cash or time fees to retrieve product price and quality information via digital platforms.

Positive cognitive search friction: Information screening generates mental fatigue, reflected by the concave search utility function:$U_{s}(s),\frac{\partial u_{s}(s)}{\partial s}>\text{0},\frac{\partial^{\text{2}}u_{s}(s)}{\partial s^{\text{2}}}<\text{0}$.

The concave functional form ensures marginal utility gains from additional search decline continuously as primitive cognitive-friction parameters shift to raise endogenous equilibrium search intensity $s^{*}$. Rational consumers balance information benefits and cognitive fatigue to select an interior optimal search intensity strictly bounded by 0<s<1. No model parameter forces this boundary exogenously; This interior equilibrium solution ${\text{s}}^{*}$ emerges endogenously from consumer utility maximization. We supplement the first‑order and second‑order optimality conditions for consumer search maximization in S1 Text.1 and A.8 to rigorously verify concavity and interior maximum. In comparative‑static analysis, ${\text{s}}^{*}$ denotes the endogenously solved equilibrium search intensity derived from consumer utility maximization. We perturb primitive cognitive‑friction parameters, which shift ${\text{s}}^{*}$, and in turn alter firms’ equilibrium advertising intensity and profitability. We avoid shorthand wording that would treat s as an exogenous primitive parameter. All demand and profit adjustments driven by this dual-cost structure are fully derived in S1 Text, Sections A7–A8.

### 3.4. The advertising

Firms select continuous advertising intensity ϕ, where the probability a consumer receives no brand ad approximates $(1-\phi )\approx e^{-N}$ under large total ad volume N. The convex advertising cost function follows standard industrial organization specifications: $A\left(\phi \right)=\lambda \phi^{2}/2$, with λ>0 representing marginal advertising expenditure.

The share of consumers conducting voluntary information search is denoted s∈[0,1]. Loyal consumers purchase from their home brand regardless of ad exposure, while comparison shoppers only transact after receiving ads or completing independent search. Period 1 exclusive demand for Firm i: $D_{i}^{1}=\alpha +\beta \phi_{i}\left(1-\phi_{j}\right);\;$cross-brand competitive demand $D_{ij}^{1}=\beta \phi_{i}\phi_{j}$. Full layered demand derivation is provided in S1 Text to avoid logical jumps.

## 4. Equilibrium Analysis of dynamic targeted advertising strategy and information search

### 4.1. Link between pure‑strategy advertising equilibrium and mixed‑strategy pricing

This subsection clarifies the linkage between first‑stage pure‑strategy advertising choices and the second‑stage mixed‑strategy pricing subgame. Equilibrium advertising intensity shapes the size of the loyal-consumer segment and contestable comparison‑shopper segment, which in turn determines the support bounds and expectation of the mixed‑strategy price distribution. To resolve the inconsistency identified in editorial comments, Equation (1) for two‑period discounted profit is revised to replace static price terms with conditional expected mixed‑strategy price $E\left[p_{i}^{\text{1}}\left(\phi^{*}\right)\right]$ and $E\left[p_{i}^{\text{2}}\left(\phi^{*}\right)\right]$. This formulation reflects that firm profits depend on the expectation of mixed‑strategy pricing outcomes conditional on symmetric equilibrium advertising intensity. Full derivation of conditional mixed‑strategy price distribution is presented in S2 Text.4. The subgame adapts Varian‑type sales‑model logic modified for targeted‑advertising exposure. We verify support interval, cumulative‑distribution function, atom conditions, indifference conditions, and that the distribution integrates to unity. All comparative‑static results in subsequent sections take endogenous variation in expected mixed‑strategy prices into account.

This section presents the core equilibrium analysis of the two-period duopoly targeted advertising game. Different from static advertising competition models in prior literature, this study incorporates endogenous consumer search and peer experience sharing spillovers across two periods, forming a dynamic strategic interaction framework. All mathematical derivations are simplified in the main text to focus on economic mechanisms and core conclusions. No explicit closed‑form analytical solution exists under this model setup. Numerical solutions are obtained by fixed‑point iteration; iteration rules and stopping criteria are documented in S2 Text.7. We obtain the complete two-period discounted total profit objective function:


$$\begin{array}{c} \Pi_{i}=\left[E\left[p_{i}^{\text{1}}\left(\phi^{*}\right)\right]\left(\alpha +\beta \phi_{i}(\text{1}-\phi_{j})\right)-\frac{\lambda }{\text{2}}\phi_{i}^{\text{2}}\right]+\delta \left\{E\left[p_{i}^{\text{2}}\left(\phi^{*}\right)\right]\left(\alpha +\beta [\phi_{i}+(\text{1}-\phi_{i})s^{*}](\text{1}-\phi_{j})(\text{1}-s^{*})\right)-\frac{\lambda }{\text{2}}\phi_{i}^{\text{2}}\right\} \end{array}$$ (1)

We solve the game via backward induction. In each period, two symmetric firms simultaneously determine targeted advertising intensity to maximize discounted two-period total profits, while pricing strategies form a mixed-strategy equilibrium in the competitive consumer segment. Consistent with the model setup, consumer information acquisition channels include official firm advertising, active pre-purchase search, and intertemporal peer experience sharing, which jointly reshape market demand and firms’ optimal advertising decisions.

Proposition 1 (Dynamic Targeted Advertising Equilibrium, Restructured).

(a) Existence: Within the constructed two-period duopoly dynamic framework integrating endogenous consumer pre-purchase search and intertemporal peer experience sharing spillovers, there exists a symmetric pure-strategy Nash equilibrium for targeted advertising intensity, proven via the Browder fixed-point theorem (S2 Text.2). S2 Text.2 explicitly verifies every prerequisite for the Browder fixed‑point theorem: non‑empty, convex compact strategy set; continuous best‑response self‑mapping; clarifies whether the object is a single‑valued best‑response function versus a correspondence.
(b) Uniqueness: Strict concavity of individual‑firm objective in its own action does not by itself guarantee uniqueness of the joint Nash equilibrium. The stationary‑point uniqueness for individual maximization is established from strict global concavity of the firm profit function with respect to advertising intensity ϕ (S2 Text.3). Strict concavity guarantees uniqueness of the stationary point but cannot exclude corner‑solution equilibria.
(c) Interiority condition: Additional explicit parameter restrictions are required to guarantee equilibrium advertising intensity $\phi^{*}$lies strictly within the open interval (0,1). When parameters violate these bounds, corner equilibria $\phi^{*}=\text{0}$ *or*${\;\phi }^{*}=\text{1}$ may emerge.

All subsequent propositions (Propositions 2–7) are conditional on interior-equilibrium constraints summarized in S2 Text.9. Existence of equilibrium does not imply uniqueness; even when existence and uniqueness hold jointly, interiority is not automatically guaranteed.

**Mechanism Explanation**. The advertising strategy set ϕ=[0,1] is non-empty, convex, and compact; each firm’s best-response mapping derived from profit maximization is continuous and self-mapping on ϕ. All three prerequisites of the Browder Fixed-Point Theorem are satisfied (full verification in S2 Text.2). Strict concavity eliminates multiple stationary points, but the unique stationary point may fall outside the feasible domain [0,1]. In such cases, the equilibrium collapses to a boundary corner solution. This proposition holds only within the interior‑equilibrium feasible parameter domain defined in S2 Text.9; Fig 2 provides a complementary numerical illustration**.**

S2 Text.1‑B.3 writes out complete first‑order and second‑order conditions including every chain‑rule derivative term arising from the dependence of $E[p_{i}^{t}(\phi^{*})]$ upon $\phi^{*}$. Concavity is proven over the relevant domain and cannot be inferred solely from the advertising‑cost‑only second‑derivative term −λ. Any envelope‑theorem simplifications are explicitly flagged in the appendices.

Full step‑by‑step first‑order condition derivation, symmetric substitution, and concavity verification for this implicit relationship are provided in S2 Text, Equations (SB‑4) to (SB‑7). All comparative static results for search intensity, sharing intensity, and advertising cost are built upon this core equilibrium expression. Full formal proof of equilibrium existence, uniqueness, and interior stability is documented in S2 Text, Subsections B2–B3. The simulation (α=0.3, β=0.4, λ=0.8, δ=0.9is visualized in Fig 2.

Solving via backward induction yields the following implicit fixed‑point relationship for symmetric interior‑equilibrium targeted‑advertising intensity $\phi^{*}$ (Equation 2). Because conditional expected mixed‑strategy prices $E[p_{i}^{t}(\phi^{*})]$ themselves depend on equilibrium advertising intensity $\phi^{*}$, this expression cannot be reduced to an explicit closed‑form solution under the present model setup. The full implicit system appears in S2 Text Equation (SB‑7).


$$\begin{array}{c} \phi^{*}=\frac{\beta \left[E\left[p_{i}^{\text{1}}\left(\phi^{*}\right)\right]+\delta E\left[p_{i}^{\text{2}}\left(\phi^{*}\right)\right](\text{1}-s^{*}{)}^{\text{2}}\right]}{\lambda +\beta \left[E\left[p_{i}^{\text{1}}\left(\phi^{*}\right)\right]+\delta E\left[p_{i}^{\text{2}}\left(\phi^{*}\right)\right](\text{1}-s^{*}{)}^{\text{2}}\right]} \end{array}$$ (2)

Full step-by-step first-order condition derivation, symmetric substitution, and concavity verification for this implicit relationship are provided in S2 Text, Equations (SB‑4) to (SB‑7). All comparative static results for search intensity, sharing intensity, and advertising cost are built upon this core equilibrium expression. Full formal proof of equilibrium existence, uniqueness, and interior stability is documented in S2 Text, Subsections B2–B3.

**Proposition 2 (Determinants and Trade-Offs of Equilibrium Advertising Investment)**. Conditional on interior‑equilibrium feasible‑parameter domain: Under the model assumptions, firms’ symmetric equilibrium targeted‑advertising intensity is increasing in the share of price‑sensitive comparison consumers and consumer reservation value v, and decreasing in advertising‑cost coefficient λ. Two‑period inter‑temporal information spillovers modify static advertising cost‑benefit trade‑offs and produce forward‑looking equilibrium adjustments. *(Proof location:* *Text S2 Sup. Derivations* S1-S3).

**Mechanism Explanation**. A larger share of price-sensitive comparison consumers intensifies market competition, enabling targeted advertising to effectively capture rival market share and generate higher marginal revenue, thereby motivating firms to increase advertising investment. A higher consumer product reservation value elevates the upper bound of product pricing, improving the profit return of advertising conversion. Conversely, higher advertising costs raise the marginal expenditure of market information coverage and suppress firms’ advertising willingness. Beyond this static trade-off relationship, the two-period dynamic framework further optimizes firms’ advertising decisions by incorporating intertemporal information spillovers, laying a solid theoretical foundation for analyzing the heterogeneous impacts of consumer search and peer sharing behaviors. This proposition holds only within the interior-equilibrium feasible parameter domain defined in S2 Text.9; Fig 2 provides a complementary numerically illustrated demonstration. Comparative static partial derivative derivations for reservation value v, comparison consumer share β, and advertising cost coefficient λ are available in S2 Text, Supplementary Derivations S1–S3.

## 5. Strategic effect of consumer search behavior and corresponding propositions

This section elaborates the economic connotations of consumer search behavior, systematically clarifying the dual differentiated effects of search intensity on firms’ targeted advertising strategies and long-term profitability. All theoretical inferences in this section are strictly anchored to mathematical derivations and numerically illustrated simulation results presented in the appendices, forming a rigorous logical chain for core mechanism analysis.

Active consumer search acts as a core endogenous information governance mechanism that shapes consumer decision-making in digital marketing ecosystems. Unlike prior studies that adopt exogenous information shock frameworks or treat consumer search as a static, passive behavioral factor, this study endogenizes search intensity as a utility-maximizing behavioral outcome. This refined setting aligns with real-world digital market characteristics, where consumers independently adjust their information search depth based on cognitive friction and information benefit trade-offs, rather than being passively affected by external information stimuli. This section systematically unpacks the causal relationship between endogenous search intensity, equilibrium targeted advertising strategies, and duopoly firms’ profitability, and clarifies the microeconomic mechanisms and boundary conditions dominating the strategic effects of consumer search.

In terms of advertising strategy adjustment, endogenous consumer search generates a significant strategic substitution effect on firms’ targeted advertising investment. From a micro-information transmission perspective, firms’ official targeted advertising is constrained by fixed coverage scopes and investment costs, inevitably leaving partial consumer groups uncovered by commercial information. In contrast, active pre-purchase consumer search serves as a spontaneous supplementary information channel, enabling consumers to acquire complete product attribute and pricing information independently, without relying on firms’ advertising delivery. As primitive cognitive‑friction parameters shift so that the endogenous equilibrium search intensity $s^{*}$ rises, the exclusive information advantage of targeted advertising is weakened continuously, leading to a sustained decline in the marginal demand contribution and marginal revenue of incremental advertising investment. To maximize long-term discounted profits and avoid inefficient competitive investment, rational duopoly firms will strategically reduce redundant targeted advertising expenditure. This substitution relationship is rigorously verified via marginal elasticity analysis and chain-rule differentiation in S1 Text and S2 Text (SB8–SB10), eliminating theoretical ambiguity regarding the correlation between consumer search and corporate advertising decisions.

In terms of market operation and profit performance, although consumer search partially substitutes for the information dissemination function of targeted advertising, it delivers a stable, sustainable profit enhancement effect for duopoly firms via market efficiency complementarity. The core driving force lies in the optimization of digital market information transparency and consumer decision-making efficiency. In low-search-intensity market scenarios, severe endogenous information asymmetry prevails: most potential consumers cannot accurately distinguish product value and competitive advantages, resulting in low purchase conversion efficiency and massive waste of corporate advertising resources. With the improvement of consumer search willingness and depth, cross-brand information barriers are broken, information friction in the product market is effectively reduced, and the matching efficiency between consumer demand and firm supply is significantly optimized, thereby substantially improving the effective conversion rate of potential market demand and corporate equilibrium profits.

Notably, the profit-promoting effect of consumer search presents obvious diminishing marginal returns, forming a typical concave growth trend of corporate equilibrium profits. At low search intensity, the market is plagued by severe information opacity, and enhanced consumer search can bring substantial marginal efficiency gains and profit growth. When search intensity rises to a high level, most market information friction has been eliminated, and the marginal improvement space of market matching efficiency is gradually compressed, resulting in a slowdown in profit growth. This concave characteristic theoretically explains the coexistence of consumer search substitution and advertising necessity in digital markets, clarifying why rational firms will not completely abandon targeted advertising even in high-search-intensity market environments.

In summary, endogenous consumer search behavior constructs a unique dual optimization mechanism of “strategic substitution + efficiency complementarity” in duopoly targeted advertising competition. On the strategic investment side, it restrains excessive homogeneous advertising competition among firms and optimizes corporate cost structure; on the market operation side, it improves overall information transparency and supply-demand matching efficiency, and continuously boosts corporate profitability. This dual-mechanism conclusion effectively supplements the deficiencies of existing advertising competition literature, which generally ignores the endogenous dynamic adjustment characteristics of consumer information behaviors and their heterogeneous impacts on firm strategic decisions.

All qualitative mechanism analyses presented above are numerically illustrated alongside formal mathematical derivations and numerical simulation results in the appendices. Based on backward induction equilibrium solutions and comparative static analysis of the two-period duopoly model (S2 Text, SB8–SB10), this study further formalizes the core strategic effects of consumer search into two rigorous theoretical propositions, which correspond consistently to all model calculation results and simulation visualization conclusions.

Proposition 3 Strategic Substitution Effect of Consumer Search on Targeted Advertising.

Conditional on interior-equilibrium feasible-parameter domain: Endogenous consumer active search behavior exerts a prominent strategic substitution effect on firms’ targeted advertising strategies. As primitive cognitive‑friction parameters shift so that endogenous equilibrium search intensity $s^{*}$ rises, independent consumer information acquisition compensates for the insufficient information coverage of official targeted advertising, reduces the marginal demand contribution and marginal revenue of corporate advertising, and ultimately drives firms to strategically cut redundant targeted advertising investment to reduce operating costs (Proof location: S2 Text, Equations (SB-8)-(SB-10)).

**Mechanism Explanation**. Corporate targeted advertising and consumer active search serve as two alternative consumer information acquisition channels in digital markets. When firms’ official advertising fails to achieve full market coverage, consumers can independently obtain product attribute and price information through active search, which weakens the marginal utility of corporate advertising. With the continuous improvement of consumer search capability (driven by shifts in primitive cognitive-friction parameters), the marginal revenue brought by incremental advertising investment declines continuously. To avoid redundant investment and resource waste, rational firms will actively reduce excessive advertising expenditure. This substitution relationship is fully verified by marginal elasticity derivation in S1 Text and intuitively reflected by the downward trend of equilibrium advertising intensity in Fig 2. This proposition holds only within the interior-equilibrium feasible parameter domain defined in S2 Text.9; Fig 2 provides a complementary numerical illustration. Full mathematical proof of the negative monotonic relationship between s and equilibrium $\phi^{*}$ is derived via chain-rule differentiation in S2 Text, Equations (SB-8)–(SB-10).

Proposition 4 (Concave-Profit-Growth Effect of Consumer Search via Efficiency Complementarity).

**Conditional on interior-equilibrium feasible-parameter domain:** Consumer active search monotonically improves duopoly firms’ two-period discounted equilibrium profits by mitigating market information asymmetry and optimizing consumer purchase decision-making efficiency. This profit-promoting effect exhibits significant diminishing marginal returns, forming a concave increasing profit trend, which systematically verifies the dual “strategic substitution + efficiency complementarity” mechanism of consumer search behavior (Proof location: S2 Text, Subsection B6.2).

**Mechanism Explanation**. Although consumer search partially substitutes the information dissemination function of targeted advertising and reduces firms’ advertising investment scale, it fundamentally optimizes market information matching efficiency. In low-search scenarios, severe information asymmetry leaves numerous potential consumers unable to accurately identify product value, resulting in low purchase conversion rates and wasted advertising resources. As search intensity increases, market information transparency is significantly enhanced, consumer decision-making efficiency is optimized, and the conversion rate of potential market demand is substantially improved. Meanwhile, the profit growth effect gradually weakens at high search levels, as most market information friction is eliminated. This concave growth characteristic, visualized by the upward and flattening curve in Fig 3, explains why consumer search can sustainably boost corporate profits without enabling firms to completely abandon targeted advertising. This proposition holds only within the interior-equilibrium feasible parameter domain defined in S2 Text.9; Fig 3 provides a complementary numerically illustrated demonstration. Proof of positive first-order derivative and negative second-order concave profit curvature with respect to search intensity $s^{*}$ is provided in S2 Text, Subsection B6.2.

## 6. Nonlinear influence of peer experience sharing and core propositions

Different from the monotonic optimization effect of consumer search, peer experience sharing (exogenous parameter (e) generated by intertemporal transaction spillovers has a dual nonlinear impact on corporate profits, forming a distinctive inverted-U relationship. This core finding effectively explains the contradictory phenomena in real digital markets: user-generated experience sharing can both activate market potential and intensify industrial competition, which cannot be captured by traditional linear analysis frameworks. The detailed micro-mechanism and mathematical threshold verification are supplemented in S1 Text and S2 Text.

At low levels of experience sharing, peer sharing behavior plays a dominant role in market value expansion. Official targeted advertising has limited coverage and can only reach a fixed group of consumers. In contrast, decentralized peer experience sharing breaks through the boundary of corporate advertising investment, spreading product quality and usage experience to potential consumer groups that are not covered by commercial advertising. This spontaneous information spillover expands overall market awareness, activates latent market demand, and brings positive spillover profits to duopoly firms. At this stage, user sharing effectively complements the deficiency of official marketing, and the improvement of sharing intensity is conducive to corporate profit growth.

When experience sharing exceeds the critical threshold, the competitive erosion effect gradually dominates and inhibits corporate profitability. With the continuous increase of exogenous sharing parameter e, the model does not endogenously generate content composition shift; profit inversion comes purely from demand‑price equilibrium feedback inside the model. Transparent large‑scale sharing significantly improves the overall price sensitivity of market consumers, weakens the brand premium space formed by targeted advertising, and intensifies homogeneous price competition between duopoly firms. Sustained intensified competition squeezes corporate profit margins, resulting in a gradual decline in equilibrium profits (Fig 4).

Based on the threshold derivation and nonlinear comparative static results in S2 Text, we formalize the core nonlinear propositions of peer experience sharing as follows.

Proposition 5 (Inverted-U Nonlinear Profit Effect of Peer Experience Sharing).

**Conditional on interior-equilibrium feasible-parameter domain:** Peer experience sharing formed by intertemporal transaction spillovers (captured by exogenous parameter e) exerts a distinctive inverted-U nonlinear impact on corporate equilibrium profits. There exists a unique critical sharing threshold $e_{crit}$ fully expressed as function of primitive‑model parameters (α,β,λ,δ,v). Below the threshold, peer sharing expands market information coverage and activates latent market demand to facilitate corporate profit growth; above the threshold, excessive peer sharing triggers intensified homogeneous price competition, erodes brand premium space, and continuously suppresses corporate profitability. If $e_{crit}$ falls outside admissible domain e∈[0,1], monotonic outcomes appear instead of inverted‑U (Proof location: S2 Text.7).

**Mechanism Explanation**. The nonlinear profit effect stems from the alternation of two opposing endogenous mechanisms of peer sharing. At low sharing intensity, decentralized user-generated experience sharing breaks the coverage limitations of official targeted advertising, disseminating product quality and usage experience to potential consumer groups unreached by commercial advertising. This spontaneous information spillover expands overall market awareness, activates latent market demand, and generates positive profit spillovers for duopoly firms, effectively compensating for the deficiencies of corporate official marketing.

Once sharing intensity exceeds the critical threshold, demand‑price equilibrium feedback dominates. Higher exogenous sharing intensity e raises consumers’ overall price sensitivity purely through model‑internal demand adjustments, weakens the brand premium formed by targeted advertising, and intensifies homogeneous price competition between duopoly firms. At this stage, the competitive erosion effect completely dominates the market expansion effect, leading to continuous profit decline. We do not infer any endogenous user‑generated‑content composition shift purely from scalar e variation. This proposition holds only within the interior‑equilibrium feasible parameter domain defined in Text S2.9; Fig 5 provides complementary numerically illustrated demonstration. Threshold derivation for critical sharing intensity $e_{crit}$ and piece‑wise marginal profit sign analysis are fully derived in S2 Text.7.

**Proposition 6 (Heterogeneous Mechanism Differences Between Dual Information Channels)**. Official targeted advertising and consumer active search are standardized, normative information transmission and acquisition mechanisms with stable monotonic market optimization effects. In contrast, decentralized, unregulated peer experience sharing (exogenous parameter e) has dual attributes of market expansion and competitive erosion, and its economic impact is strictly dependent on spillover intensity, resulting in essential differences from the two standardized information mechanisms.

**Mechanism Explanation**. Both corporate targeted advertising and consumer active search are standardized behavioral mechanisms that stabilize market information order and optimize consumer decision-making efficiency in all market scenarios. In comparison, peer experience sharing lacks standardized guidance and supervision: moderate sharing supplements market information and expands demand, while excessive sharing induces heightened homogeneous price competition. This fundamental difference makes peer sharing a double-edged sword for digital marketing, reasonably explaining the coexistence of “market empowerment and heightened competitive pressure” in real digital markets. This proposition holds only within the interior‑equilibrium feasible parameter domain defined in S2 Text.9. Comparative static contrast between standardized search/advertising channels and decentralized peer sharing is formalized in S1 Text, Subsection A9.

## 7. Interactive Mechanism and Strategic Equilibrium Optimization with Dual Propositions

This study clarifies the interactive mechanism between search intensity and experience sharing intensity. Consumer search moderates the utilization efficiency of existing peer sharing information rather than generating sharing-related behaviors. Cross-partial derivative results demonstrate that search intensity solely exerts a moderating influence on the market economic effects of peer information spillovers, which eliminates unreasonable causal associations between consumer search intensity s and experience sharing intensity e.

Consumer search and peer experience sharing, as two core information channels in digital markets, do not independently affect firms’ advertising strategies and profit performance. There exists a significant asymmetric interaction between the two information mechanisms, which jointly determine the dynamic evolution of market competition structure and firms’ optimal strategic choices. This section further analyzes the interactive logic of dual information channels and summarizes optimized advertising strategies for different market scenarios, forming a complete theoretical and strategic closed loop of the study.

The essential difference between the two information channels determines their asymmetric interaction relationship. Consumer active search is a rational information screening behavior initiated by consumers themselves, which always optimizes market information efficiency and improves decision-making accuracy, belonging to a single positive efficiency mechanism. In contrast, peer experience sharing is a passive information spillover behavior with dual attributes of benefit and risk, whose economic effect depends entirely on the intensity of spillover. The superposition of the two heterogeneous mechanisms produces state contingent interaction effects. Mathematically, the non zero cross partial derivative $\frac{\partial^{2}\Pi^{*}}{\partial s^{*}\partial e}\neq 0$ validates the existence of interaction effects between the two information channels as derived in S2 Text.8.

At low levels of exogenous sharing intensity e, consumer search and peer experience sharing act as complementary forces. Consumer search improves consumers’ ability to digest peer generated content, amplifying the market expansion benefits brought by moderate peer sharing. Under this market condition, the joint effect of the two information channels can expand overall market awareness without triggering excessive price competition, creating favorable profit conditions for duopoly firms. Correspondingly, firms can appropriately reduce redundant targeted advertising spending given improved consumer information access.

At high levels of exogenous sharing intensity e, consumer search amplifies the competitive erosion effect of peer sharing. Because higher search ability enables consumers to quickly locate and compare price information embedded within peer reviews, homogeneous price competition becomes more severe, which erodes firm profit margins.

Proposition 7 (State-contingent interactive effects between Search and Peer-experience sharing).

**Conditional on interior-equilibrium feasible-parameter domain**: The marginal profit influence of endogenous equilibrium search intensity $s^{*}$ *d*epends on the magnitude of exogenous peer‑experience sharing parameter e. When sharing intensity stays below the critical threshold $e_{crit}$, search amplifies the profit enhancing market expansion effect of peer sharing; when sharing intensity exceeds the critical threshold, higher search intensity aggravates competitive price erosion originating from peer generated price information (Proof location: S2 Text.8).

**Mechanism Explanation**. Consumer search does not generate peer sharing content by itself; instead, it improves consumer utilization of already existing peer information pools. At low e, peer content is dominated by quality related product introductions, and enhanced consumer search helps more consumers access this positive information and expand market demand. At high e, peer content contains abundant cross‑brand price comparison information. Improved search capability accelerates consumers’ price comparison activities and intensifies duopoly price competition. This proposition holds only within the interior equilibrium feasible parameter domain defined in S2 Text.9.

Managerial Implication (qualitative inference beyond formal model propositions; no mathematical proof).

The baseline model does not formally endogenize advertising content differentiation between quality‑focused versus price‑focused messages. The following statement represents a cautious practical inference derived from our equilibrium results, rather than a mathematically proven formal proposition. Modelling advertising message content constitutes a promising avenue for future theoretical research.

**Mechanism Explanation**. In high-search and low-sharing markets, consumer active search and moderate peer sharing jointly undertake market information coverage functions, and excessive corporate advertising will lead to information redundancy and cost waste. Appropriately reducing advertising investment can effectively optimize cost structure while maintaining stable market awareness and market share. In high-search and high-sharing markets, the superposition of dual information spillovers intensifies homogeneous price competition and squeezes profit margins. Traditional price-oriented advertising can only trigger continuous competitive pressure. Firms may consider differentiated brand‑ and quality‑oriented advertising to weaken consumers’ price sensitivity and build brand premium advantages. This reasoning is tentative and requires future empirical investigation. Boundary conditions are explored within the examined parameter range in S2 Text, Subsection B.9.

## 8. Discussion

### 8.1. Theoretical contributions

This paper constructs a symmetric two‑period duopoly dynamic game model that incorporates consumer pre‑purchase search (generating endogenous equilibrium search intensity $s^{*}$) and post‑purchase peer‑experience sharing (modelled via the exogenous symmetric parameter e). Seven formal theoretical propositions receive mathematical proof in S1 Text and S2 Text; their behavior is examined via sensitivity analysis within the examined parameter range.

Qualitative interpretive commentary (non‑formal‑proposition):From model equilibrium outputs, targeted advertising and consumer search function as information‑processing mechanisms within this model setting. Exogenous peer sharing can produce both beneficial spillover and competitive‑erosion outcomes depending on parameter‑value realizations. This is qualitative observation and is not a mathematically proven formal proposition.

Our theoretical contributions are three‑fold. First, we integrate pre‑purchase consumer search and post‑purchase peer‑experience sharing within a unified duopoly targeted advertising framework. Most existing literature studies these two consumer information behaviors in isolation, whereas we reveal their asymmetric complementary‑competitive interaction effects on advertising and profit outcomes. Second, we revise the conventional search cost assumption by separating zero monetary access cost from positive cognitive friction, which better matches digital market conditions and generates endogenous interior search equilibrium $s^{*}$. Third, we unpack the dual effect of peer‑experience sharing and identify its inverted‑U shaped profit relationship, and we further demonstrate that the economic impact of sharing is moderated by endogenous consumer search intensity. All model predictions apply only under stated model assumptions and within the feasible interior equilibrium parameter domain defined in S2 Text.9.

### 8.2. Sensitivity analysis and robustness examination

All numerical outputs presented in figures serve purely as numerically illustrated examples rather than empirical validation. Full sensitivity tests traverse economically meaningful ranges for key structural parameters, including advertising cost coefficient λ, comparison consumer fraction β, and inter‑temporal discount factor δ. Supplementary S1‑S3 Figs visualise these robustness checks. Monotonic trends, concave properties, and the inverted‑U non‑linear relationship remain qualitatively stable across parameter variations. Analytical derivations in S2 Text represent the primary formal basis for all propositions.

### 8.3. Practical managerial and marketing implications

All illustrative industry examples below serve solely as contextual illustration to frame abstract theoretical mechanisms. This study is a purely analytical game‑theoretic model without empirical data collection or econometric testing. No real‑world firm behavior constitutes empirical validation for model predictions.

(1) Adjust targeted advertising intensity with respect to underlying drivers of consumer search propensity

Based on Propositions 3‑4 and numerical illustrations, firms may adjust advertising intensity when market‑level primitive cognitive‑friction parameters shift endogenous equilibrium search intensity $s^{*}$. As a contextual illustration only, product categories where consumers exhibit high information‑seeking tendencies may permit reduced redundant targeted‑advertising expenditure, whereas categories with low consumer information‑seeking tendencies may require broader promotional reach. This parallel is provided for illustration and does not represent empirically validated causal guidance.

(2) Anticipate nonlinear consequences of peer experience sharing

The model predicts an inverted‑U relationship between exogenous sharing intensity e and equilibrium profit within the feasible interior‑equilibrium parameter domain. Because sharing intensity e is treated as exogenous in this baseline model, we cannot derive precise operational thresholds for firm‑managed sharing interventions. The model suggests that peer‑generated content may expand market awareness at low levels, yet amplify price‑comparison pressures at higher levels. Tentatively, this points toward potential benefits of avoiding heavy price disclosure within targeted‑advertising messages; shifting emphasis toward quality attributes may represent a promising direction for future empirical study.

(3) Adopt forward-looking dynamic perspective for digital markets

Under the two‑period model’s dynamic spillover structure, firms may benefit from monitoring changes in consumer information environments over time. Contextually, brand operators often track peer‑generated content trends via social‑listening tools. This real‑world practice is presented purely as contextual illustration and is not derived formally from model optimization.

Beyond the model‑derived comparative‑static results, our theoretical framework also yields interpretive insights regarding corporate strategic resilience. Dynamic targeted‑advertising decisions must continuously adapt to evolving consumer information‑search and peer‑sharing behaviors; firms lacking such strategic flexibility may suffer profit erosion when market information conditions shift. This observation aligns with recent discussions of adaptive and resilient business‑model design [24]. We emphasize that this corporate‑resilience reading constitutes a qualitative interpretation of our game‑theoretic outputs rather than a formally proven proposition.

### 8.4. Research limitations and future research directions

This study has several important limitations that open paths for subsequent research. (1)The model assumes symmetric firms and homogeneous products; equilibrium outcomes may change under asymmetric‑cost structures or differentiated‑product quality. (2) Sharing intensity e is exogenous scalar for analytical tractability; the model does not endogenise sharing generation or content composition. (3) We rely on fully rational agents; bounded‑rationality behavioral specifications may alter equilibrium comparative‑static patterns. (4) Our main formal results focus upon interior‑parameter‑domain equilibria; some conclusions may fail under corner‑solution equilibria ($\phi^{*}=\text{0}$ or $\phi^{*}=\text{1}$). (5) Advertising message‑content differentiation (quality versus price focus), review incentive design, and complaint management systems are not endogenized within our formal model setup. Accordingly, any suggestions touching these areas should be treated as hypotheses awaiting future empirical research rather than model‑derived conclusions. (6) Numerical sensitivity over selected‑parameter windows does not establish general empirical robustness or real‑world generalizability. Simulation figures serve for illustrative purposes only.

Future work could endogenize sharing behavior, allowing firms or consumers to influence the magnitude of peer spillovers. Empirical studies using market panel data could test whether the model‑predicted inverted‑U pattern between peer sharing and firm profit is observable in real digital markets. Finally, future models may incorporate multi‑period infinite‑horizon settings to investigate long‑run evolution of consumer information ecosystems, advertising exposure supervision, and platform algorithm recommendation rules [25] to explore the evolutionary logic of targeted advertising strategies under institutional constraints, forming a more comprehensive and practical digital marketing competition theory [26].

## 9. Conclusion

This paper develops a two‑period symmetric duopoly game model to investigate how pre‑purchase consumer information search and post‑purchase peer‑experience sharing jointly shape firms’ targeted‑advertising strategies and equilibrium profit performance in digital markets. We distinguish s as generic consumer search choice variable, $s^{*}$ as endogenous equilibrium search intensity determined by primitive cognitive‑friction parameters, and e as an exogenous symmetric peer‑experience sharing parameter. Seven formal mathematical propositions are derived and proven within the feasible interior‑equilibrium parameter domain documented in S2 Text.9.

Our core findings can be summarised as follows. First, as primitive cognitive‑friction parameters shift to raise endogenous equilibrium search intensity $s^{*}$, firms reduce their equilibrium targeted‑advertising intensity, yet firm profits rise in a concave increasing fashion because consumer search improves overall market information efficiency. Second, peer‑experience sharing produces an inverted‑U effect on firm profits: moderate sharing expands market awareness and boosts profit, but beyond a critical threshold excessive sharing intensifies homogeneous price competition and suppresses profit levels. Third, there exists state contingent interaction between search and sharing. At low sharing levels search amplifies profit gains from peer information spillovers; at high sharing levels search amplifies competitive price erosion. A qualitative managerial implication (without formal mathematical proof) suggests that firms may adjust advertising content orientation according to market information conditions, though this insight requires future empirical validation.

All model‑derived predictions hold only under the stated modelling assumptions and within the feasible interior‑equilibrium parameter domain defined in S2 Text.9. Numerical simulations provided in main text figures and supplementary materials are numerically illustrated and do not constitute empirical validation. This study advances targeted advertising theory by jointly modelling two key consumer information behaviors under zero monetary search cost with cognitive friction assumption, and offers tentative neutral theoretical insights for digital marketing practitioners. We hope that the theoretical predictions presented here can stimulate future empirical and theoretical extensions in this research domain.

## Supporting information

S1 Text Consumer Utility, Demand Structure, and Full Two-Period Derivation. This file includes subsections A1 to A10 with additional detailed proofs of consumer utility, demand structure, and full two-period derivation.
(DOCX)

S2 Text Complete Nash Equilibrium Proof, Analytical Equilibrium Solution, Full Comparative Statics and Robustness Analysis. This file includes subsections B1 to B12 with additional detailed descriptions of the complete Nash Equilibrium proof, analytical equilibrium solution, full comparative statics and robustness analysis.
(DOCX)

S3 Text MATLAB Code for numerical simulations and computational analysis. This supporting‑information document contains the replication read‑me note and five MATLAB source scripts: params_config.m, calc_Ep.m, solve_phi_eq.m, main_simulation.m, and plot_all_figures.m. Implementation instructions, computational environment, and baseline parameter settings are documented within this file.
(DOCX)

S1 FigSensitivity to advertising cost λ∈0.4,0.8,1.2.(TIF)

S2 FigSensitivity to comparison‑shopper share β∈0.1,0.3,0.5.(TIF)

S3 FigSensitivity to discount factor δ∈0.2,0.5,0.8.(TIF)
