# Supplementary material for: The complementary-competitive interplay of consumer information search and experience sharing: Implications for targeted advertising and firm performance
Source: PLoS One. 2026 Oct 28;21(10):e0340849. doi: 10.1371/journal.pone.0340849 (PMC13716209; doi:10.1371/journal.pone.0340849)
Supplement: Supplementary file 1 [file pone.0340849.s001.docx]

***Appendix A.*** **Consumer Utility, Demand Structure, and Full Two-Period Derivation**

**A1. Model Primitives and Consumer Utility Maximization**

This study develops a symmetric two-period duopoly game framework to characterize firms’ targeted advertising strategies in digital markets with endogenous consumer information acquisition. All agents in the model are fully rational: firms maximize discounted two-period profits, while consumers make purchase decisions based on utility maximization. The entire market size is normalized to one, and no exogenous stochastic shocks or behavioral biases are introduced, ensuring a clean analytical setup for game-theoretic equilibrium analysis.

All consumers share identical product valuation and face the same market information environment. Their purchase utility is jointly determined by product price, inherent product value, and information-based utility gains generated by active search and peer experience sharing. The generalized consumer utility function is specified as follows:

$U=v-p+u_{s}(s)+u_{e}(e)$  (*SA*-1)

where $v$ denotes the homogeneous consumer reservation value for the product, representing the maximum willingness to pay;$p$ is the actual transaction price; $u_{s}(s)$ captures the utility improvement brought by voluntary pre-purchase information search; and $u_{e}(e)$ reflects the utility adjustment induced by peer post-purchase experience sharing.

Important definitional note: $s$ denotes the generic consumer choice variable for search effort; $s^{*}=arg\max_{s}U(s;\theta)$ is the endogenous equilibrium search intensity solved from consumer utility maximization. $\theta$ stands for primitive cognitive‑friction structural parameters that determine $s^{*}$. Comparative‑static analysis perturbs primitive cognitive‑friction parameters inside $u_{s}(\cdot)$, rather than directly shifting $s^{*}$. Experience‑sharing intensity $e$ is an exogenous symmetric primitive scalar parameter. The baseline model contains only scalar $e$; it does not formally endogenise mapping from purchase volume to sharing quantity or content‑composition shifts.

Consistent with the characteristics of online consumer search behavior, this model adopts a zero monetary access price, with non‑negligible cognitive or effort friction assumption. In digital scenarios, consumers can browse product information without explicit monetary payment, but information screening and comparison still consume cognitive resources, resulting in incomplete information elimination. Functionally, $u_{s}(s)$ is strictly increasing and strictly concave in search intensity $s$, which captures diminishing marginal benefits of information search and guarantees interior equilibrium search intensity within $s\in(0,1)$. **Consumer search intensity** $s^{*}$ **is an equilibrium solution from utility maximization, not an exogenous parameter. Comparative‑static analysis perturbs primitive cognitive‑friction parameters inside** $u_{s}(.)$ **, rather than directly shifting** $s^{*}$**.**

**Consumer search first‑order / second‑order optimality conditions and corner‑solution check**

Consumers choose search intensity $s$ to maximize their net search‑related utility $u_{s}(s)$ , subject to feasible domain $s\in[0,1]$ .

First‑order necessary condition for interior optimum:$\begin{matrix} \frac{\partial u_{s}(s)}{\partial s}=0 \end{matrix}$

Second‑order sufficient condition for interior maximum:
$\begin{matrix} \frac{\partial^{2}u_{s}(s)}{\partial s^{2}}<0 \end{matrix}$

The strict concavity of $u_{s}(\cdot)$ guarantees that any stationary point satisfying (SA‑1‑1) constitutes a local maximum. We further evaluate the two boundary corner candidates $s=0$ and $s=1$ :

At $s=0$ : marginal utility of additional search satisfies $\partial u_{s}/\partial s|_{s=0}>0$ , meaning raising search intensity improves consumer utility; therefore $s=0$ cannot be an optimal choice. Corner checks confirm $s=0$ and $s=1$ yield sub‑optimal utility under admissible primitive parameters. Interior optimum (0 < $s^{*}$<1) emerges endogenously from utility maximization without ad‑hoc external constraints.

A consumer completes a purchase if and only if the overall utility is non-negative ($U\geq0$). In the baseline equilibrium analysis, the core purchasing constraint simplifies to $p\leq v$, while the heterogeneous information effects of search and experience sharing are fully embedded in the dynamic two-period demand adjustment process.

**A2. Consumer Segmentation and Market Structure Constraint**

To characterize differentiated consumer responses to advertising and information spillover, the market is divided into two mutually-exclusive and exhaustive consumer groups: brand-loyal consumers and price-sensitive comparison consumers. This segmentation setting is widely adopted in oligopoly advertising competition research and effectively captures the coexistence of brand stickiness and market competition in real industries.

1. **Loyal consumers**: Each firm possesses a fixed proportion $\alpha$ of loyal consumers. These consumers have stable brand preferences and only purchase products from their incumbent brand, regardless of rival firms’ advertising intensity and pricing strategies. This segment forms the stable captive market share of each duopoly firm; loyal consumers remain fully captive up to reservation value $v$.

2. **Comparison consumers**: The remaining market consists of $\beta$ price-sensitive consumers. This group does not have fixed brand preferences and will actively collect advertising information, conduct price comparisons, and refer to peer sharing content to make purchase decisions, constituting the fully competitive segment of the market.

The model satisfies strict market closure and normalization constraints to ensure complete market coverage without overlapping or missing segments:

$2\alpha+\beta=1,\alpha=\frac{1-\beta}{2}$	(*SA*-2)

This symmetric market structure eliminates endogenous market heterogeneity between the two firms, ensuring that all equilibrium differences are solely derived from information channel effects rather than initial market endowment gaps.

**3. Peer‑sharing utility term** $u_{e}(e)$**:**

The sharing intensity parameter $e$ is treated as exogenous symmetric primitive variable to maintain analytical tractability. The term $u_{e}(e)$ captures utility for Period‑2 consumers reading peer reviews. Period‑1 consumers generating peer content obtain zero direct utility from writing reviews; peer sharing is an uncompensated inter‑temporal spillover. Search effort $s$ changes how efficiently consumers utilize existing peer‑sharing information pool and does not generate sharing volume itself. The model does not endogenize content‑composition transformation purely from scalar variation in $e$.

**A3. Advertising Exposure Probability and Standard Advertising Cost Function**

In the duopoly targeted advertising game, both firms independently and endogenously determine continuous targeted advertising intensity $\phi_{i}\in[0,1]$ ($i=A,B$). The advertising intensity is defined as the probability that a randomly selected consumer in the market receives valid and recognizable targeted advertising from firm $i$. Correspondingly, $1-\phi_{i}$ represents the probability that consumers fail to obtain official advertising information from the focal firm.

To conform to mainstream industrial organization theory and guarantee the mathematical tractability of equilibrium solutions, this study employs a standard strictly convex quadratic advertising cost function. This functional form features increasing marginal advertising costs, which effectively avoids extreme corner solutions (zero or full advertising investment) and ensures the existence and uniqueness of interior optimal advertising intensity.

The formal advertising cost function is specified as:

$C(\phi_{i})=\frac{\lambda}{2}\phi_{i}^{2},\lambda>0$	(*SA*-3)

where $\lambda$ is the exogenous advertising cost coefficient. A larger $\lambda$ implies higher marginal expenditure for firms to improve advertising coverage, which restricts excessive advertising competition in the duopoly market. Under the symmetric duopoly framework, Firm A and Firm B have identical cost structures, so heterogeneous cost interference in strategic equilibrium is completely excluded.

**Mutually‑exhaustive demand decomposition, unit‑market‑mass conservation algebra, tie‑breaking allocation rules for mixed‑strategy outcomes.**

Total market mass is normalized to unity. All consumer groups are disjoint and fully partition the whole market; no double‑counting or omitted population. Under mixed‑strategy pricing subgame, tie‑breaking rule: when firms draw exactly equal prices, comparison shoppers are randomly split equally between the two duopoly firms. Algebraic summation of period‑1 and period‑2 aggregated market demand always equals 1 under feasible parameter domain. Loyal consumer demand is invariant to mixed‑strategy price draws as long as $p\leq v$.

**A4. Period 1 Full Demand Derivation (No Intertemporal Information Spillover)**

The first period represents the initial stage of the dynamic game, where no prior transaction records or peer experience sharing exists in the market. Consumer information acquisition channels are limited to two types: firms’ official targeted advertising and consumers’ voluntary pre-purchase information search. No intertemporal information spillover occurs, and the market information environment only depends on current-period corporate advertising behavior.

For any firm $i$, total market demand consists of two independent components: fixed loyal consumer demand and variable comparison consumer demand. The loyal consumer segment generates stable base demand $\alpha$, which is completely insensitive to advertising competition and price adjustments.

For the comparison consumer segment with total proportion $\beta$, purchase decisions depend entirely on bilateral advertising exposure status of the two firms, which can be divided into two mutually exclusive scenarios:

First, **single-brand advertising exposure**: comparison consumers only receive advertising from firm $i$ but not firm $j$, with a occurrence probability of $\phi_{i}(1-\phi_{j})$. In this case, consumers confirm product value through voluntary search and exclusively purchase products from firm $i$, forming non-competitive exclusive demand.

Second, **dual-brand advertising exposure**: comparison consumers receive advertising from both firms simultaneously, with a occurrence probability of $\phi_{i}\phi_{j}$. Consumers in this scenario will conduct cross-brand price and quality comparison, forming a fully competitive market segment where firms compete for market share through pricing strategies.

Based on probability weighting and strict market segmentation rules, the **Period 1 exclusive demand** of firm $i$ is derived as:

$D_{i}^{1}=\alpha+\beta\phi_{i}(1-\phi_{j})$  (*SA*-4)

The **Period 1 cross-brand competitive demand** of firm $i$ is derived as:

$D_{ij}^{1}=\beta\phi_{i}\phi_{j}$	(*SA*-5)

All Period 1 demand formulas strictly satisfy market clearing conditions, with no repeated calculation or missing market segments, and are fully consistent with consumer utility maximization logic.

**A5. Period 2 Full Demand Derivation (Including Experience Sharing Spillover & Search Correction)**

Different from the static information environment of Period 1, Period 2 features endogenous intertemporal information spillover. Consumers’ information acquisition channels are expanded to three dimensions: firm targeted advertising, voluntary pre-purchase information search, and peer post-purchase experience sharing generated from Period 1 transactions. Exogenous scalar parameter $e$ enters Period‑2 utility, then feeds into Period‑2 demand structure and subsequently firm profit; full mapping is laid out in utility function SA‑1 and demand below. The joint effect of search intensity $s$ and experience sharing intensity$e$ fundamentally corrects market information asymmetry and reshapes the dynamic demand structure of duopoly firms.

Voluntary consumer search effectively compensates for the insufficient coverage of official targeted advertising. Even if consumers do not receive advertising from the focal firm, they can still actively obtain product information through online search behavior. We define the effective comprehensive information coverage rate of firm $i$ after incorporating consumer search as:

$\phi_{i}+(1-\phi_{i})s$	(*SA*-6)

Notably, consumer search cannot completely eliminate market information friction. To retain the strategic competition space of duopoly firms and conform to real market information incompleteness, we set $1-s$ as the residual information uncertainty coefficient after search behavior. This setting avoids the extreme scenario of complete market information transparency and ensures the rationality of corporate strategic adjustment. Demand expressions are consistent with revised profit function Equation (1), which uses conditional expected mixed‑strategy prices $E\left[ p\left( \phi^{*} \right) \right]$ in place of static price values.

Combined with the intertemporal spillover characteristics of experience sharing (sharing enhances consumer price sensitivity and strengthens cross-brand comparison motivation), we strictly derive the Period 2 exclusive demand of firm $i$:

$D_{i}^{2}=\alpha+\beta[\phi_{i}+(1-\phi_{i})s](1-\phi_{j})(1-s)$	(*SA*-7)

Correspondingly, the Period 2 cross-brand competitive demand formed by dual-firm information coverage is specified as:

$D_{ij}^{2}=\beta[\phi_{i}+(1-\phi_{i})s][\phi_{j}+(1-\phi_{j})s]$	(*SA*-8)

Experience sharing intensity $e$ acts on Period 2 market demand through implicit endogenous spillover mechanisms rather than direct exogenous variables. The model does not infer content‑composition shifts purely from scalar $e$ variation. Moderate peer sharing expands overall market information transparency, improves consumer product recognition, and increases effective demand conversion rate; excessive price-oriented peer sharing will intensify heightened homogeneous price competition, squeeze firms’ exclusive demand, and expand competitive market segments. This dual endogenous effect lays a solid theoretical foundation for the inverted-U profit relationship of experience sharing proposed in the core propositions.

**A6. Two-Period Discounted Total Profit Function Full Derivation**

Firms’ operating revenue derives from product sales to loyal and comparison consumers, while operating costs are solely composed of convex targeted advertising investment costs incurred in each separate period. Consistent with standard dynamic game assumptions, forward-looking firms do not maximize single-period static profit but pursue the maximization of discounted total profit over two periods. Let $\delta\in(0,1]$ denote the intertemporal discount factor, which measures firms’ valuation of future profits; a larger $\delta$ indicates that firms pay more attention to long-term market performance.

The single-period profit of firm $i$ in period $t$ equals total sales revenue minus current advertising investment cost, with the basic expression:

$\pi_{i}^{t}=p_{i}^{t}D_{i}^{t}-\frac{\lambda}{2}\phi_{i}^{2},t=1,2$  (*SA*-9)

where $p_{i}^{t}$ represents the equilibrium transaction price of firm $i$ in period $t$, and $D_{i}^{t}$ is the total effective market demand of the focal firm in the current period. Substituting the Period 1 and Period 2 demand functions derived above, we obtain the complete two-period discounted total profit objective function:

$\Pi_{i}=\left[ p_{i}^{1}\left( \alpha+\beta\phi_{i}(1-\phi_{j}) \right)-\frac{\lambda}{2}\phi_{i}^{2} \right]+\delta\left[ p_{i}^{2}\left( \alpha+\beta(\phi_{i}+(1-\phi_{i})s)(1-\phi_{j})(1-s) \right)-\frac{\lambda}{2}\phi_{i}^{2} \right]$	(*SA*-10)

This profit function has good mathematical properties: it is continuously differentiable in the domain of advertising intensity and strictly concave in the firm’s own advertising strategy. No exogenous fixed profit terms or artificial constraints are added, ensuring standard and rigorous game-theoretic optimization analysis.

Note: Main‑text Equation (1) replaces static price terms with conditional expected mixed‑strategy price $E[p_{i}^{t}(\phi^{*})]$ for mixed‑strategy pricing subgame, see Appendix B.4**.**

**A7. Advertising Demand Elasticity Derivation (Period 1 & Period 2)**

To clarify the marginal economic mechanism of targeted advertising and identify the substitution relationship between consumer search and corporate advertising, this section derives the first-order marginal demand effect of advertising intensity in two periods respectively.

In Period 1, without search and sharing interference, the marginal demand contribution of firm$i$’s advertising intensity is:

$\frac{\partial D_{i}^{1}}{\partial\phi_{i}}=\beta(1-\phi_{j})$  ( *SA*-10)

This result indicates that the marginal effectiveness of a firm’s advertising is negatively affected by the rival’s advertising intensity. When competitors increase targeted advertising coverage ($\phi_{j}\uparrow$), the blank market space that can be captured by unilateral advertising shrinks, and the marginal revenue of the focal firm’s incremental advertising investment decreases accordingly.

In Period 2, active consumer search changes the information structure of the market and further adjusts the marginal effect of advertising. The marginal demand contribution of advertising intensity is revised to:

$\frac{\partial D_{i}^{2}}{\partial\phi_{i}}=\beta(1-s)^{2}(1-\phi_{j})$	( *SA*-11)

The embedded $(1-s)^{2}$ term clearly verifies the substitution effect of consumer search on advertising. Higher search intensity improves consumer information acquisition efficiency, reduces the incremental demand brought by official advertising, and fundamentally weakens firms’ incentive for excessive advertising investment.

**A8. Micro-Mechanism of Search-Advertising Complementarity**

When conducting comparative statics, we vary underlying cognitive‑friction primitives. Chain‑rule logic applies: primitive parameters $\to$ shift in $s^{*}\to$change in equilibrium advertising $\phi^{*}$ and total profit $\Pi^{*}$. Direct exogenous manipulation of$s^{*}$is avoided throughout the analysis.

Although consumer information search partially replaces the information coverage function of targeted advertising and reduces the marginal effectiveness of advertising, it generates a net complementary profit-enhancing effect by optimizing the overall market information environment. This complementary mechanism mainly operates through two microeconomic channels.

First, consumer search eliminates invalid information asymmetry caused by insufficient official advertising coverage. In the absence of search behavior, consumers who are not reached by advertising cannot identify product value, resulting in potential market loss. Active search makes up for the deficiency of corporate advertising, improves consumer trust and purchase conversion rate, and reduces firms’ invalid advertising investment waste.

Second, search optimizes market matching efficiency and stabilizes corporate market share. Consumers with high search intensity can make more rational purchase decisions, reduce blind brand switching caused by information scarcity, and weaken the volatility of competitive market demand. This stable market environment further improves firms’ long-term profitability.

As shown in Appendix A.1, interior search optimum $s^{*}\in(0,1)$ emerges endogenously from utility maximization; corner outcomes $s=0$ and $s=1$ are suboptimal under admissible parameters. Exogenously fixing $s$ to boundary values would violate consumer utility‑maximizing behavior.

In summary, consumer search does not simply crowd out advertising value; it optimizes the efficiency of the entire market information system, reduces marginal advertising costs, and ultimately raises firms’ equilibrium profit level, which theoretically explains the monotonic positive correlation between search intensity and corporate profit.

**A9. Micro-Mechanism of Experience Sharing Dual Effect**

Different from the single positive efficiency improvement effect of information search, peer experience sharing has dual opposing effects on firm performance, which jointly generate the core inverted-U nonlinear profit relationship in this study. The dominant effect of sharing behavior depends entirely on the intensity of peer spillover.

At low experience sharing levels, user-generated content mainly plays the role of expanding market information coverage. A large number of consumers who are not covered by official advertising can understand product quality and value through peer sharing, which expands potential market demand, improves overall product awareness, and brings significant positive profit spillover to firms.

At high experience sharing levels, profit inversion originates purely from demand‑price equilibrium feedback within the model; we do not infer structural content‑composition change purely from scalar $e$ variation. Large-scale transparent peer sharing disseminates cross-brand price information, significantly enhances the price sensitivity of comparison-shoppers, weakens the brand premium space formed by targeted advertising, and intensifies homogeneous price competition between duopoly firms. At this stage, the competitive erosion effect completely dominates, resulting in declining corporate profits.

This endogenous dual mechanism of experience sharing constitutes the core theoretical innovation of this study and provides a solid micro-foundation for the subsequent proposition derivation and numerical simulation results.

**A10. Model Boundary and Assumption Consistency Verification**

All derivations in Appendix A strictly follow the unified model axiom system, including symmetric duopoly firms, homogeneous product quality, zero marginal production cost, zero monetary search cost with cognitive friction, dual consumer segmentation, and two-period dynamic information spillover. All demand and profit functions satisfy four core consistency constraints.

First, strict market clearing: the total market demand of the two firms always normalizes to one, ensuring complete market coverage without structural deviation. Second, non-negative equilibrium solution: all demand and profit values in the equilibrium domain are economically non-negative, avoiding invalid negative economic returns. Third, profit function strict concavity: guaranteeing unique interior optimal solutions for corporate strategic decisions. Fourth, cross-period logical consistency: the information spillover logic of the two-period dynamic game is self-consistent without contradictory assumptions.

The entire analytical system does not contain any ad-hoc empirical restrictions or contradictory theoretical settings, meeting the rigorous logical requirements of peer review.
