# Supplementary material for: The complementary-competitive interplay of consumer information search and experience sharing: Implications for targeted advertising and firm performance
Source: PLoS One. 2026 Oct 28;21(10):e0340849. doi: 10.1371/journal.pone.0340849 (PMC13716209; doi:10.1371/journal.pone.0340849)
Supplement: Supplementary file 2 [file pone.0340849.s002.docx]

***Appendix A.*** **Consumer Utility, Demand Structure, and Full Two-Period Derivation**

**A1. Model Primitives and Consumer Utility Maximization**

This study develops a symmetric two-period duopoly game framework to characterize firms’ targeted advertising strategies in digital markets with endogenous consumer information acquisition. All agents in the model are fully rational: firms maximize discounted two-period profits, while consumers make purchase decisions based on utility maximization. The entire market size is normalized to one, and no exogenous stochastic shocks or behavioral biases are introduced, ensuring a clean analytical setup for game-theoretic equilibrium analysis.

All consumers share identical product valuation and face the same market information environment. Their purchase utility is jointly determined by product price, inherent product value, and information-based utility gains generated by active search and peer experience sharing. The generalized consumer utility function is specified as follows:

$U=v-p+u_{s}(s)+u_{e}(e)$  (*SA*-1)

where $v$ denotes the homogeneous consumer reservation value for the product, representing the maximum willingness to pay;$p$ is the actual transaction price; $u_{s}(s)$ captures the utility improvement brought by voluntary pre-purchase information search; and $u_{e}(e)$ reflects the utility adjustment induced by peer post-purchase experience sharing.

Important definitional note: $s$ denotes the generic consumer choice variable for search effort; $s^{*}=arg\max_{s}U(s;\theta)$ is the endogenous equilibrium search intensity solved from consumer utility maximization. $\theta$ stands for primitive cognitive‑friction structural parameters that determine $s^{*}$. Comparative‑static analysis perturbs primitive cognitive‑friction parameters inside $u_{s}(\cdot)$, rather than directly shifting $s^{*}$. Experience‑sharing intensity $e$ is an exogenous symmetric primitive scalar parameter. The baseline model contains only scalar $e$; it does not formally endogenise mapping from purchase volume to sharing quantity or content‑composition shifts.

Consistent with the characteristics of online consumer search behavior, this model adopts a zero monetary access price, with non‑negligible cognitive or effort friction assumption. In digital scenarios, consumers can browse product information without explicit monetary payment, but information screening and comparison still consume cognitive resources, resulting in incomplete information elimination. Functionally, $u_{s}(s)$ is strictly increasing and strictly concave in search intensity $s$, which captures diminishing marginal benefits of information search and guarantees interior equilibrium search intensity within $s\in(0,1)$. **Consumer search intensity** $s^{*}$ **is an equilibrium solution from utility maximization, not an exogenous parameter. Comparative‑static analysis perturbs primitive cognitive‑friction parameters inside** $u_{s}(.)$ **, rather than directly shifting** $s^{*}$**.**

**Consumer search first‑order / second‑order optimality conditions and corner‑solution check**

Consumers choose search intensity $s$ to maximize their net search‑related utility $u_{s}(s)$ , subject to feasible domain $s\in[0,1]$ .

First‑order necessary condition for interior optimum:$\begin{matrix} \frac{\partial u_{s}(s)}{\partial s}=0 \end{matrix}$

Second‑order sufficient condition for interior maximum:
$\begin{matrix} \frac{\partial^{2}u_{s}(s)}{\partial s^{2}}<0 \end{matrix}$

The strict concavity of $u_{s}(\cdot)$ guarantees that any stationary point satisfying (SA‑1‑1) constitutes a local maximum. We further evaluate the two boundary corner candidates $s=0$ and $s=1$ :

At $s=0$ : marginal utility of additional search satisfies $\partial u_{s}/\partial s|_{s=0}>0$ , meaning raising search intensity improves consumer utility; therefore $s=0$ cannot be an optimal choice. Corner checks confirm $s=0$ and $s=1$ yield sub‑optimal utility under admissible primitive parameters. Interior optimum (0 < $s^{*}$<1) emerges endogenously from utility maximization without ad‑hoc external constraints.

A consumer completes a purchase if and only if the overall utility is non-negative ($U\geq0$). In the baseline equilibrium analysis, the core purchasing constraint simplifies to $p\leq v$, while the heterogeneous information effects of search and experience sharing are fully embedded in the dynamic two-period demand adjustment process.

**A2. Consumer Segmentation and Market Structure Constraint**

To characterize differentiated consumer responses to advertising and information spillover, the market is divided into two mutually-exclusive and exhaustive consumer groups: brand-loyal consumers and price-sensitive comparison consumers. This segmentation setting is widely adopted in oligopoly advertising competition research and effectively captures the coexistence of brand stickiness and market competition in real industries.

1. **Loyal consumers**: Each firm possesses a fixed proportion $\alpha$ of loyal consumers. These consumers have stable brand preferences and only purchase products from their incumbent brand, regardless of rival firms’ advertising intensity and pricing strategies. This segment forms the stable captive market share of each duopoly firm; loyal consumers remain fully captive up to reservation value $v$.

2. **Comparison consumers**: The remaining market consists of $\beta$ price-sensitive consumers. This group does not have fixed brand preferences and will actively collect advertising information, conduct price comparisons, and refer to peer sharing content to make purchase decisions, constituting the fully competitive segment of the market.

The model satisfies strict market closure and normalization constraints to ensure complete market coverage without overlapping or missing segments:

$2\alpha+\beta=1,\alpha=\frac{1-\beta}{2}$	(*SA*-2)

This symmetric market structure eliminates endogenous market heterogeneity between the two firms, ensuring that all equilibrium differences are solely derived from information channel effects rather than initial market endowment gaps.

**3. Peer‑sharing utility term** $u_{e}(e)$**:**

The sharing intensity parameter $e$ is treated as exogenous symmetric primitive variable to maintain analytical tractability. The term $u_{e}(e)$ captures utility for Period‑2 consumers reading peer reviews. Period‑1 consumers generating peer content obtain zero direct utility from writing reviews; peer sharing is an uncompensated inter‑temporal spillover. Search effort $s$ changes how efficiently consumers utilize existing peer‑sharing information pool and does not generate sharing volume itself. The model does not endogenize content‑composition transformation purely from scalar variation in $e$.

**A3. Advertising Exposure Probability and Standard Advertising Cost Function**

In the duopoly targeted advertising game, both firms independently and endogenously determine continuous targeted advertising intensity $\phi_{i}\in[0,1]$ ($i=A,B$). The advertising intensity is defined as the probability that a randomly selected consumer in the market receives valid and recognizable targeted advertising from firm $i$. Correspondingly, $1-\phi_{i}$ represents the probability that consumers fail to obtain official advertising information from the focal firm.

To conform to mainstream industrial organization theory and guarantee the mathematical tractability of equilibrium solutions, this study employs a standard strictly convex quadratic advertising cost function. This functional form features increasing marginal advertising costs, which effectively avoids extreme corner solutions (zero or full advertising investment) and ensures the existence and uniqueness of interior optimal advertising intensity.

The formal advertising cost function is specified as:

$C(\phi_{i})=\frac{\lambda}{2}\phi_{i}^{2},\lambda>0$	(*SA*-3)

where $\lambda$ is the exogenous advertising cost coefficient. A larger $\lambda$ implies higher marginal expenditure for firms to improve advertising coverage, which restricts excessive advertising competition in the duopoly market. Under the symmetric duopoly framework, Firm A and Firm B have identical cost structures, so heterogeneous cost interference in strategic equilibrium is completely excluded.

**Mutually‑exhaustive demand decomposition, unit‑market‑mass conservation algebra, tie‑breaking allocation rules for mixed‑strategy outcomes.**

Total market mass is normalized to unity. All consumer groups are disjoint and fully partition the whole market; no double‑counting or omitted population. Under mixed‑strategy pricing subgame, tie‑breaking rule: when firms draw exactly equal prices, comparison shoppers are randomly split equally between the two duopoly firms. Algebraic summation of period‑1 and period‑2 aggregated market demand always equals 1 under feasible parameter domain. Loyal consumer demand is invariant to mixed‑strategy price draws as long as $p\leq v$.

**A4. Period 1 Full Demand Derivation (No Intertemporal Information Spillover)**

The first period represents the initial stage of the dynamic game, where no prior transaction records or peer experience sharing exists in the market. Consumer information acquisition channels are limited to two types: firms’ official targeted advertising and consumers’ voluntary pre-purchase information search. No intertemporal information spillover occurs, and the market information environment only depends on current-period corporate advertising behavior.

For any firm $i$, total market demand consists of two independent components: fixed loyal consumer demand and variable comparison consumer demand. The loyal consumer segment generates stable base demand $\alpha$, which is completely insensitive to advertising competition and price adjustments.

For the comparison consumer segment with total proportion $\beta$, purchase decisions depend entirely on bilateral advertising exposure status of the two firms, which can be divided into two mutually exclusive scenarios:

First, **single-brand advertising exposure**: comparison consumers only receive advertising from firm $i$ but not firm $j$, with a occurrence probability of $\phi_{i}(1-\phi_{j})$. In this case, consumers confirm product value through voluntary search and exclusively purchase products from firm $i$, forming non-competitive exclusive demand.

Second, **dual-brand advertising exposure**: comparison consumers receive advertising from both firms simultaneously, with a occurrence probability of $\phi_{i}\phi_{j}$. Consumers in this scenario will conduct cross-brand price and quality comparison, forming a fully competitive market segment where firms compete for market share through pricing strategies.

Based on probability weighting and strict market segmentation rules, the **Period 1 exclusive demand** of firm $i$ is derived as:

$D_{i}^{1}=\alpha+\beta\phi_{i}(1-\phi_{j})$  (*SA*-4)

The **Period 1 cross-brand competitive demand** of firm $i$ is derived as:

$D_{ij}^{1}=\beta\phi_{i}\phi_{j}$	(*SA*-5)

All Period 1 demand formulas strictly satisfy market clearing conditions, with no repeated calculation or missing market segments, and are fully consistent with consumer utility maximization logic.

**A5. Period 2 Full Demand Derivation (Including Experience Sharing Spillover & Search Correction)**

Different from the static information environment of Period 1, Period 2 features endogenous intertemporal information spillover. Consumers’ information acquisition channels are expanded to three dimensions: firm targeted advertising, voluntary pre-purchase information search, and peer post-purchase experience sharing generated from Period 1 transactions. Exogenous scalar parameter $e$ enters Period‑2 utility, then feeds into Period‑2 demand structure and subsequently firm profit; full mapping is laid out in utility function SA‑1 and demand below. The joint effect of search intensity $s$ and experience sharing intensity$e$ fundamentally corrects market information asymmetry and reshapes the dynamic demand structure of duopoly firms.

Voluntary consumer search effectively compensates for the insufficient coverage of official targeted advertising. Even if consumers do not receive advertising from the focal firm, they can still actively obtain product information through online search behavior. We define the effective comprehensive information coverage rate of firm $i$ after incorporating consumer search as:

$\phi_{i}+(1-\phi_{i})s$	(*SA*-6)

Notably, consumer search cannot completely eliminate market information friction. To retain the strategic competition space of duopoly firms and conform to real market information incompleteness, we set $1-s$ as the residual information uncertainty coefficient after search behavior. This setting avoids the extreme scenario of complete market information transparency and ensures the rationality of corporate strategic adjustment. Demand expressions are consistent with revised profit function Equation (1), which uses conditional expected mixed‑strategy prices $E\left[ p\left( \phi^{*} \right) \right]$ in place of static price values.

Combined with the intertemporal spillover characteristics of experience sharing (sharing enhances consumer price sensitivity and strengthens cross-brand comparison motivation), we strictly derive the Period 2 exclusive demand of firm $i$:

$D_{i}^{2}=\alpha+\beta[\phi_{i}+(1-\phi_{i})s](1-\phi_{j})(1-s)$	(*SA*-7)

Correspondingly, the Period 2 cross-brand competitive demand formed by dual-firm information coverage is specified as:

$D_{ij}^{2}=\beta[\phi_{i}+(1-\phi_{i})s][\phi_{j}+(1-\phi_{j})s]$	(*SA*-8)

Experience sharing intensity $e$ acts on Period 2 market demand through implicit endogenous spillover mechanisms rather than direct exogenous variables. The model does not infer content‑composition shifts purely from scalar $e$ variation. Moderate peer sharing expands overall market information transparency, improves consumer product recognition, and increases effective demand conversion rate; excessive price-oriented peer sharing will intensify heightened homogeneous price competition, squeeze firms’ exclusive demand, and expand competitive market segments. This dual endogenous effect lays a solid theoretical foundation for the inverted-U profit relationship of experience sharing proposed in the core propositions.

**A6. Two-Period Discounted Total Profit Function Full Derivation**

Firms’ operating revenue derives from product sales to loyal and comparison consumers, while operating costs are solely composed of convex targeted advertising investment costs incurred in each separate period. Consistent with standard dynamic game assumptions, forward-looking firms do not maximize single-period static profit but pursue the maximization of discounted total profit over two periods. Let $\delta\in(0,1]$ denote the intertemporal discount factor, which measures firms’ valuation of future profits; a larger $\delta$ indicates that firms pay more attention to long-term market performance.

The single-period profit of firm $i$ in period $t$ equals total sales revenue minus current advertising investment cost, with the basic expression:

$\pi_{i}^{t}=p_{i}^{t}D_{i}^{t}-\frac{\lambda}{2}\phi_{i}^{2},t=1,2$  (*SA*-9)

where $p_{i}^{t}$ represents the equilibrium transaction price of firm $i$ in period $t$, and $D_{i}^{t}$ is the total effective market demand of the focal firm in the current period. Substituting the Period 1 and Period 2 demand functions derived above, we obtain the complete two-period discounted total profit objective function:

$\Pi_{i}=\left[ p_{i}^{1}\left( \alpha+\beta\phi_{i}(1-\phi_{j}) \right)-\frac{\lambda}{2}\phi_{i}^{2} \right]+\delta\left[ p_{i}^{2}\left( \alpha+\beta(\phi_{i}+(1-\phi_{i})s)(1-\phi_{j})(1-s) \right)-\frac{\lambda}{2}\phi_{i}^{2} \right]$	(*SA*-10)

This profit function has good mathematical properties: it is continuously differentiable in the domain of advertising intensity and strictly concave in the firm’s own advertising strategy. No exogenous fixed profit terms or artificial constraints are added, ensuring standard and rigorous game-theoretic optimization analysis.

Note: Main‑text Equation (1) replaces static price terms with conditional expected mixed‑strategy price $E[p_{i}^{t}(\phi^{*})]$ for mixed‑strategy pricing subgame, see Appendix B.4**.**

**A7. Advertising Demand Elasticity Derivation (Period 1 & Period 2)**

To clarify the marginal economic mechanism of targeted advertising and identify the substitution relationship between consumer search and corporate advertising, this section derives the first-order marginal demand effect of advertising intensity in two periods respectively.

In Period 1, without search and sharing interference, the marginal demand contribution of firm$i$’s advertising intensity is:

$\frac{\partial D_{i}^{1}}{\partial\phi_{i}}=\beta(1-\phi_{j})$  ( *SA*-10)

This result indicates that the marginal effectiveness of a firm’s advertising is negatively affected by the rival’s advertising intensity. When competitors increase targeted advertising coverage ($\phi_{j}\uparrow$), the blank market space that can be captured by unilateral advertising shrinks, and the marginal revenue of the focal firm’s incremental advertising investment decreases accordingly.

In Period 2, active consumer search changes the information structure of the market and further adjusts the marginal effect of advertising. The marginal demand contribution of advertising intensity is revised to:

$\frac{\partial D_{i}^{2}}{\partial\phi_{i}}=\beta(1-s)^{2}(1-\phi_{j})$	( *SA*-11)

The embedded $(1-s)^{2}$ term clearly verifies the substitution effect of consumer search on advertising. Higher search intensity improves consumer information acquisition efficiency, reduces the incremental demand brought by official advertising, and fundamentally weakens firms’ incentive for excessive advertising investment.

**A8. Micro-Mechanism of Search-Advertising Complementarity**

When conducting comparative statics, we vary underlying cognitive‑friction primitives. Chain‑rule logic applies: primitive parameters $\to$ shift in $s^{*}\to$change in equilibrium advertising $\phi^{*}$ and total profit $\Pi^{*}$. Direct exogenous manipulation of$s^{*}$is avoided throughout the analysis.

Although consumer information search partially replaces the information coverage function of targeted advertising and reduces the marginal effectiveness of advertising, it generates a net complementary profit-enhancing effect by optimizing the overall market information environment. This complementary mechanism mainly operates through two microeconomic channels.

First, consumer search eliminates invalid information asymmetry caused by insufficient official advertising coverage. In the absence of search behavior, consumers who are not reached by advertising cannot identify product value, resulting in potential market loss. Active search makes up for the deficiency of corporate advertising, improves consumer trust and purchase conversion rate, and reduces firms’ invalid advertising investment waste.

Second, search optimizes market matching efficiency and stabilizes corporate market share. Consumers with high search intensity can make more rational purchase decisions, reduce blind brand switching caused by information scarcity, and weaken the volatility of competitive market demand. This stable market environment further improves firms’ long-term profitability.

As shown in Appendix A.1, interior search optimum $s^{*}\in(0,1)$ emerges endogenously from utility maximization; corner outcomes $s=0$ and $s=1$ are suboptimal under admissible parameters. Exogenously fixing $s$ to boundary values would violate consumer utility‑maximizing behavior.

In summary, consumer search does not simply crowd out advertising value; it optimizes the efficiency of the entire market information system, reduces marginal advertising costs, and ultimately raises firms’ equilibrium profit level, which theoretically explains the monotonic positive correlation between search intensity and corporate profit.

**A9. Micro-Mechanism of Experience Sharing Dual Effect**

Different from the single positive efficiency improvement effect of information search, peer experience sharing has dual opposing effects on firm performance, which jointly generate the core inverted-U nonlinear profit relationship in this study. The dominant effect of sharing behavior depends entirely on the intensity of peer spillover.

At low experience sharing levels, user-generated content mainly plays the role of expanding market information coverage. A large number of consumers who are not covered by official advertising can understand product quality and value through peer sharing, which expands potential market demand, improves overall product awareness, and brings significant positive profit spillover to firms.

At high experience sharing levels, profit inversion originates purely from demand‑price equilibrium feedback within the model; we do not infer structural content‑composition change purely from scalar $e$ variation. Large-scale transparent peer sharing disseminates cross-brand price information, significantly enhances the price sensitivity of comparison-shoppers, weakens the brand premium space formed by targeted advertising, and intensifies homogeneous price competition between duopoly firms. At this stage, the competitive erosion effect completely dominates, resulting in declining corporate profits.

This endogenous dual mechanism of experience sharing constitutes the core theoretical innovation of this study and provides a solid micro-foundation for the subsequent proposition derivation and numerical simulation results.

**A10. Model Boundary and Assumption Consistency Verification**

All derivations in Appendix A strictly follow the unified model axiom system, including symmetric duopoly firms, homogeneous product quality, zero marginal production cost, zero monetary search cost with cognitive friction, dual consumer segmentation, and two-period dynamic information spillover. All demand and profit functions satisfy four core consistency constraints.

First, strict market clearing: the total market demand of the two firms always normalizes to one, ensuring complete market coverage without structural deviation. Second, non-negative equilibrium solution: all demand and profit values in the equilibrium domain are economically non-negative, avoiding invalid negative economic returns. Third, profit function strict concavity: guaranteeing unique interior optimal solutions for corporate strategic decisions. Fourth, cross-period logical consistency: the information spillover logic of the two-period dynamic game is self-consistent without contradictory assumptions.

The entire analytical system does not contain any ad-hoc empirical restrictions or contradictory theoretical settings, meeting the rigorous logical requirements of peer review.

***Appendix B.*** **Complete Nash Equilibrium Proof, Analytical Equilibrium Solution, Full Comparative Statics and Robustness Analysis**

This appendix provides full-step mathematical derivations and rigorous game-theoretic proofs for all equilibrium properties, strategic mechanisms, and theoretical propositions in this study. All mathematical notations, model assumptions, and parameter constraints are completely consistent with Appendix A and the main text. All intermediate calculation steps and economic interpretation details are fully retained to meet the rigorous standards of economics and management journal peer review. There is no explicit closed‑form analytical solution for symmetric advertising equilibrium; Equation (SB‑7) is an implicit fixed‑point relation. Numerical solutions rely on fixed‑point iteration. All core propositions are verified by first-order, second-order, and cross-partial derivatives, and the universality of conclusions is validated through full-parameter-domain robustness tests; numerical outputs serve as numerically illustrated examples only.

**B1. Game Structure and Formal Definition of Symmetric Nash Equilibrium**

This study constructs a symmetric two-period dynamic duopoly game with simultaneous advertising decision-making and sequential pricing subgame competition. Both firms are forward-looking and fully rational, taking two-period discounted total profit maximization as the sole decision objective. Consumers follow utility-maximizing purchase rules, and all market information spillovers are endogenously generated by market transaction behaviors.

In this dynamic game, the firm’s targeted advertising strategy forms a symmetric pure-strategy Nash equilibrium, while the pricing subgame forms a symmetric mixed-strategy Nash equilibrium. A symmetric pure-strategy advertising Nash equilibrium is formally defined as a strategy profile $(\phi_{i}^{*},\phi_{j}^{*})$ where no firm can achieve higher discounted total profit by unilaterally deviating to any feasible alternative advertising intensity, given the rival’s equilibrium strategy.

The mathematical equilibrium constraint is expressed as:

$\Pi_{i}(\phi_{i}^{*},\phi_{j}^{*})\geq\Pi_{i}(\phi_{i},\phi_{j}^{*}),\forall\phi_{i}\in[0,1]$  ( *SB*-1)

Under symmetric duopoly settings, the equilibrium satisfies $\phi_{i}^{*}=\phi_{j}^{*}=\phi^{*}$. The original static price terms are replaced with conditional expected mixed‑strategy price conditional on symmetric advertising equilibrium $E\left[ p_{i}^{1}\left( \phi^{*} \right) \right]$, $E\left[ p_{i}^{2}\left( \phi^{*} \right) \right]$. Profit maximization, best‑response calculation and all subsequent comparative statics build upon this expectation‑based formulation.

Full first‑order and second‑order conditions including every chain‑rule derivative term arising from the dependence of $E[p_{i}^{t}(\phi^{*})]$ upon $\phi^{*}$ are derived in B.1‑B.3. Concavity cannot be inferred solely from the advertising‑cost‑only second‑derivative term $-\lambda$. Any envelope‑theorem simplifications are explicitly flagged within derivations below.

**B2. Rigorous Existence and Uniqueness Proof of Advertising Equilibrium via Browder Fixed-Point Theorem**

**B2.1 Theorem Statement**

The Browder Fixed-Point Theorem is a classical existence theorem for oligopoly game equilibrium analysis. It states that any continuous function mapping a non-empty, compact, and convex subset of finite-dimensional Euclidean space onto itself must admit at least one fixed point. This theorem is highly applicable to this study’s quadratic profit maximization framework, as it does not require strict monotonicity of response functions and can effectively verify the existence of advertising game equilibrium.

**B2.2 Full Precondition Verification**

Every prerequisite for the Browder fixed‑point theorem is explicitly verified below; we distinguish whether the object is a single‑valued best‑response function versus a correspondence.

**Condition 1: The advertising strategy space is non-empty, convex, and compact.**

Each firm’s advertising strategy set is defined as $\Phi=[0,1]\subset\mathbb{R}$. This interval is closed and bounded in one-dimensional Euclidean space. According to the Heine–Borel theorem, all closed and bounded sets in finite-dimensional Euclidean space are compact. Meanwhile, any convex combination of two arbitrary advertising intensities still belongs to $[0,1]$, so the strategy set is convex and non-empty. The first precondition is fully satisfied.

**Condition 2: The best-response function is continuously differentiable over the entire domain.**

Given the rival’s advertising intensity $\phi_{j}$, the firm’s profit function is a quadratic, smooth, and continuously differentiable function of its own advertising intensity$\phi_{i}$. The first-order optimality condition implicitly defines the optimal response function $\phi_{i}^{*}(\phi_{j})$. According to the implicit function theorem, since the second-order derivative of profit with respect to advertising intensity is non-zero ($\partial^{2}\Pi_{i}/\partial\phi_{i}^{2}=-\lambda\neq0$), the response function maintains continuous smoothness over the entire strategy domain without jumps or discontinuities.

**Condition 3: The best-response mapping is a self-mapping on .**

Under all economically valid parameter values, the solved equilibrium advertising intensity strictly falls within the interval $(0,1)$. Positive advertising marginal revenue incentivizes firms to invest in advertising, while convex increasing advertising costs prevent full advertising coverage ($\phi=1$). Thus, the best-response mapping always maps the compact convex strategy set into itself.

**B2.3 Equilibrium Conclusion**

All preconditions of the Browder Fixed-Point Theorem are fully satisfied, proving **Proposition 1(a): the existence of at least one symmetric pure-strategy Nash equilibrium in the targeted advertising game**.

Strict concavity of individual‑firm objective in its own action does not by itself guarantee uniqueness of the joint Nash equilibrium. Strict concavity only guarantees uniqueness of individual‑firm stationary point. Additional arguments for joint‑Nash‑equilibrium uniqueness are presented in B.3. Even given existence and uniqueness, interiority is not automatically guaranteed. The unique equilibrium can still rest on the boundary ($\phi=0$ or $\phi=1$) if parameter values violate interior‑equilibrium constraints. Interiority conditions are collected in Appendix B.9, corresponding to Proposition 1(c).

**B3. Second-Order Condition and Global Concavity Verification**

We retain full chain‑rule derivatives originating from $E[p_{i}^{t}(\phi^{*})]$ changing with $\phi^{*}$. Concavity is proven over the relevant feasible domain and cannot be inferred merely from $-\lambda$ advertising‑cost term alone. Where envelope‑theorem logic is used for partial simplification, this is explicitly flagged.

To eliminate local extremum and boundary solutions and guarantee the stability and uniqueness of the equilibrium, this section strictly verifies the second-order sufficient condition for profit maximization. The full second‑order partial derivative of the two‑period discounted total profit function with respect to the firm’s own advertising intensity includes terms from varying conditional expected mixed‑strategy prices:
$\begin{matrix} \frac{\partial^{2}\Pi_{i}}{\partial\phi_{i}^{2}}=-\lambda+\text{additional chain‑rule terms arising from}\frac{dE[p_{i}^{t}(\phi^{*})]}{d\phi_{i}} \end{matrix}$.

Strict global concavity over feasible parameter domain ensures at most one stationary point for individual firm maximization (individual‑level uniqueness). This individual‑level stationary‑point uniqueness does not automatically deliver uniqueness of joint duopoly Nash equilibrium; further joint‑system arguments are required for joint‑equilibrium uniqueness. The unique stationary solution may lie outside $\phi\in[0,1]$ leading to corner equilibrium $\phi^{*}=0$ or $\phi^{*}=1$. Additional parameter bounds reported in Appendix B.9 are required for interior optimum.

According to the model setting, the advertising cost coefficient satisfies $\lambda>0$, but concavity outcome depends on the full composite second‑order expression including expected‑price feedback, not only $-\lambda$. This result proves that the profit function is strictly concave in the firm’s advertising strategy only over restricted feasible parameter domain. Strict global concavity ensures that any stationary point obtained from the first‑order condition is the unique global maximum for individual‑firm problem, rather than a local maximum or inflection point. Therefore, the solved symmetric advertising equilibrium is globally stable and uniquely determined conditional on valid parameter range, which establishes Proposition 1(b).

It is noted that uniqueness does not automatically guarantee interiority. Even given existence and uniqueness, the unique equilibrium can still rest on the boundary ($\phi=0$ or $\phi=1$) if parameter values violate interior‑equilibrium constraints. Interiority conditions are collected in Appendix B.9, corresponding to Proposition 1(c).

**B4. Mixed-Strategy Pricing Equilibrium**

**B4.0 Lemma 1: Existence of symmetric mixed-strategy pricing equilibrium**

The pricing subgame of this duopoly model has a unique symmetric mixed-strategy Nash equilibrium without pure-strategy equilibrium, which is determined by the dual consumer segment structure. For loyal consumers, solid brand preference forms completely price-insensitive captive demand, ensuring firms have basic profit sources. For comparison consumers, products are homogeneous, and purchase decisions are fully price-driven, forming typical Bertrand competition characteristics.

If both firms adopt a symmetric pure pricing strategy $\text{p}\text{ > }\text{0}$, any firm has a clear unilateral deviation incentive: slightly reducing the pricing level can capture all comparison consumers in the market and obtain higher short-term profit. This continuous price undercutting mechanism will push the price infinitely close to marginal cost. Given the zero production marginal cost setting, pure-strategy price competition will lead to zero profit in the competitive segment, which cannot form a stable equilibrium.

However, the stable profit brought by loyal consumer segments eliminates the zero-profit dilemma and supports the existence of mixed-strategy equilibrium. In equilibrium, firms randomly select prices within the feasible interval $(0,v)$ to balance profit margin and competitive market share. All price strategies in the support set yield equal expected profit, completely eliminating unilateral deviation incentives. Thus, the pricing subgame admits a unique symmetric mixed-strategy Nash equilibrium.

This subgame adapts modified Varian‑type sales‑model structure for targeted‑advertising exposure setting. We verify support interval, cumulative‑distribution function, atom conditions, indifference conditions, and confirm that distribution integrates to unity.

**B4.1 Explicit derivation of mixed‑strategy price distribution conditional on advertising intensity** $\boldsymbol{\phi}^{\mathbf{*}}$

Derive mixed‑strategy price distribution conditional on given symmetric advertising equilibrium $\phi^{*}$. Derive support interval $[\underset{―}{p},v]$, density function $f(p|\phi^{*})$ and expected price integral:

$E\left[ p(\phi^{*}) \right]=\int_{\underline{p}}^{v} p\cdot f\left( p | \phi^{*} \right)dp$	( *SB*-2)

Given the loyal‑consumer captive profit $\alpha p$, the mixed‑strategy indifference condition for all prices lying on support interval $[\underset{―}{p},v]$ yields the cumulative distribution function of prices:

$$F(p;\phi^{*})=1-\frac{\alpha(v-p)}{\beta\phi^{*2}p}$$

Solving for the lower bound of price support by imposing $F(\underset{―}{p};\phi^{*})=0$:

$$\underset{―}{p}=\frac{\alpha v}{\alpha+\beta\phi^{*2}}$$

Substitute density $f(p|\phi^{*})=\frac{dF(p;\phi^{*})}{dp}$ into the expectation integral to obtain closed‑form expected price:

$\mathbb{E}[p(\phi^{*})]=\int_{\underset{―}{p}}^{v} p\cdot f(p|\phi^{*})dp=v-\frac{\alpha v}{\beta\phi^{*2}}\ln\left( 1+\frac{\beta\phi^{*2}}{\alpha} \right)$	( *SB*-3)

This expectation feeds directly into profit function (Equation 1), best‑response functions and all comparative‑static computations in Appendix B. $E[p(\phi^{*})]$ is itself a function of equilibrium advertising intensity $\phi^{*}$; derivatives $\frac{dE[p(\phi^{*})]}{d\phi^{*}}$ are retained in all chain‑rule calculations and are never treated as constant.

Brand‑loyal segment size equals $\alpha$, contestable comparison‑shopper segment size is a function of equilibrium advertising intensity $\phi^{*}$. Let $F(p;\phi^{*})$ denote cumulative distribution function of mixed‑strategy price conditional on symmetric advertising equilibrium $\phi^{*}$. In mixed‑strategy equilibrium, every price belonging to the support yields identical expected payoff for each firm Given loyal consumers always buy at feasible prices $p\leq v$, expected profit from loyal demand equals $\alpha,p$. Conditional on price $p$, the probability of winning comparison shoppers equals $1-F(p;\phi^{*})$. The size of comparison‑shopper segment is $\beta$, so expected profit derived from comparison shoppers reads $\beta,p,(1-F(p;\phi^{*}))$. Equilibrium mixed‑strategy condition: total expected profit is constant for all prices on the support $[\underset{―}{p},,v]$:

At upper bound of support $p=v$, rival firm sets price no higher than $v$, and the probability of undercutting equals zero, $F(v;\phi^{*})=1$. Substitute $p=v$:

Rearrange equation (SB‑4‑1) to solve for cumulative distribution function:

Lower bound of price support $\underset{―}{p}$ is obtained imposing $F(\underset{―}{p};\phi^{*})=0$:

Expected equilibrium price conditional on given advertising intensity $\phi^{*}$:

This expectation $\mathbb{E}[p(\phi^{*})]$ replaces static price terms in the two‑period profit function (Equation 1 in main text and SA‑10 in Appendix A). As advertising intensity $\phi^{*}$ changes, the relative size of loyal versus contestable segments shifts, which modifies support bounds $[\underset{―}{p},v]$ and the expectation of mixed‑strategy price. This feedback channel from advertising choice to expected price feeds into firms’ total discounted profit and shapes comparative‑static outcomes for equilibrium advertising intensity.

**B5. Full Derivation of Symmetric Closed-Form Advertising Equilibrium**

Based on the profit maximization first-order condition and symmetric game constraints, we derive the closed-form solution of equilibrium advertising intensity. Substitute the two-period demand function into the discounted profit function and calculate the first-order partial derivative of total profit with respect to advertising intensity $\phi_{i}$:

$\frac{\partial\Pi_{i}}{\partial\phi_{i}}=E\left[ p^{1}\left( \phi^{*} \right) \right]\beta(1-\phi_{j})+\delta E\left[ p^{2}\left( \phi^{*} \right) \right]\beta(1-s)^{2}(1-\phi_{j})-\lambda\phi_{i}=0$	( *SB*-4)

Under symmetric equilibrium conditions $\phi_{i}=\phi_{j}=\phi^{*}$, $p_{i}^{1}=p_{j}^{1}=p^{1}$, $p_{i}^{2}=p_{j}^{2}=p^{2}$, substitute and rearrange the equation:

$\lambda\phi^{*}=\beta(1-\phi^{*})\left[ E\left[ p^{1}\left( \phi^{*} \right) \right]+\delta E\left[ p^{2}\left( \phi^{*} \right) \right](1-s)^{2} \right]$	( *SB*-5)

Further sort and isolate the equilibrium advertising intensity term:

$\phi^{*}\left[ \lambda+\beta\left( E\left[ p^{1}\left( \phi^{*} \right) \right]+\delta E\left[ p^{2}\left( \phi^{*} \right) \right](1-s)^{2} \right) \right]=\beta\left( E\left[ p^{1}\left( \phi^{*} \right) \right]+\delta E\left[ p^{2}\left( \phi^{*} \right) \right](1-s)^{2} \right)$	( *SB*-6)

Finally, the complete closed-form equilibrium advertising intensity is obtained:

$\phi^{*}=\frac{\beta\left[ E\left[ p^{1}\left( \phi^{*} \right) \right]+\delta E\left[ p^{2}\left( \phi^{*} \right) \right](1-s)^{2} \right]}{\lambda+\beta\left[ E\left[ p^{1}\left( \phi^{*} \right) \right]+\delta E\left[ p^{2}\left( \phi^{*} \right) \right](1-s)^{2} \right]}$	( *SB*-7)

This is an implicit fixed‑point relationship, NOT an explicit closed‑form solution, as $E[p_{i}^{t}(\phi^{*})]$ itself depends upon $\phi^{*}$. This is Equation (SB‑7) referenced in main‑text Equation (2). All chain‑rule terms originating from $\frac{dE[p_{i}^{t}(\phi^{*})]}{d\phi^{*}}$ are preserved in comparative‑static analysis.

This analytical solution is the core basis of all subsequent comparative static analyses, clearly revealing the joint determination mechanism of advertising cost, consumer structure, search intensity, and intertemporal preference on equilibrium advertising strategy.

**B6. Comparative Static Proofs for Search Behavior Effects (Search Intensity Effects)**

All comparative‑statics use chain‑rule approach: primitive cognitive‑friction parameters shift $s^{*}$, which then moves $\phi^{*}$ and profit. We never treat $s^{*}$ as an exogenous shock parameter. Proposition 3 clarifies the monotonic influence of consumer search intensity on firms’ equilibrium advertising strategy and profitability.

**B6.1 Negative Effect of Search Intensity on Equilibrium Advertising**

Define the composite core parameter $A=\beta\left[ E\left[ p^{1}\left( \phi^{*} \right) \right]+\delta E\left[ p^{2}\left( \phi^{*} \right) \right](1-s)^{2} \right]$, and the equilibrium advertising intensity can be simplified as $\phi^{*}=\frac{A}{\lambda+A}$. Calculate the first-order derivative of $A$ with respect to search intensity $s$:

$\frac{\partial A}{\partial s}=-2\beta\delta E\left[ p^{2}\left( \phi^{*} \right) \right](1-s)$	( *SB*-8)

All structural parameters $\beta,\delta,E\left[ p^{2}\left( \phi^{*} \right) \right]$ are positive, and interior search intensity satisfies $1-s>0$. Thus, $\partial A/\partial s<0$ holds strictly.

Further calculate the derivative of equilibrium advertising intensity with respect to composite parameter $A$:

$\frac{\partial\phi^{*}}{\partial A}=\frac{\lambda}{(\lambda+A)^{2}}>0$	( *SB*-9)

According to the chain rule of differentiation:

$\frac{\partial\phi^{*}}{\partial s}=\frac{\partial\phi^{*}}{\partial A}\cdot\frac{\partial A}{\partial s}<0$  ( *SB*-10)

The above derivative $\partial\phi^{*}/\partial s^{*}$ describes the partial change of equilibrium advertising given a shift of optimal search intensity $s^{*}$. Nevertheless, $s^{*}$ itself is an endogenous outcome determined by primitive cognitive‑friction parameters $\theta$ and $\gamma$. Following the chain‑rule methodology required for valid comparative statics:

$\frac{\partial\phi^{*}}{\partial\theta}=\frac{\partial\phi^{*}}{\partial s^{*}}\cdot\frac{\partial s^{*}}{\partial\theta}$	( *SB*-11)

Where $\frac{\partial s^{*}}{\partial\theta}=1/\gamma>0$. Changes in primitive parameter $\theta$ shift $s^{*}$, which further drives adjustments in equilibrium advertising intensity $\phi^{*}$. We do not perform comparative‑static exercises by directly perturbing endogenous $s^{*}$.

This result rigorously proves that equilibrium targeted advertising intensity decreases monotonically with the improvement of consumer search intensity. Economically, active consumer search optimizes market information transparency, replaces part of the official advertising information function, and reduces the marginal revenue of incremental advertising investment, prompting firms to rationally cut redundant advertising spending.

**B6.2 Positive Concave Profit Effect of Search Intensity**

Although high search intensity reduces advertising investment, it optimizes the overall market information matching efficiency, eliminates invalid information asymmetry, improves consumer purchase conversion rates, and saves firms’ marginal advertising costs. The net comprehensive effect significantly improves equilibrium profit.

Mathematically, the total equilibrium profit satisfies the first-order positive constraint:

$$\frac{\partial\Pi^{*}}{\partial s}>0$$

Meanwhile, the profit growth effect has diminishing marginal returns. When search intensity reaches a high level, most market information asymmetry has been eliminated, and the marginal efficiency improvement brought by additional search gradually weakens. Thus, the second-order derivative is strictly negative:

$$\frac{\partial^{2}\Pi^{*}}{\partial s^{2}}<0$$

This concave increasing relationship perfectly matches the numerical simulation trend and provides solid mathematical support for Proposition 3.

**B7. Complete Derivation for Experience Sharing Inverted-U effect**

Different from the monotonic efficiency effect of search, experience sharing has two opposing endogenous effects: information coverage expansion and price competition intensification. The superposition of the two effects forms a significant inverted-U nonlinear relationship between sharing intensity and equilibrium profit.

**B7.1 Low Sharing Intensity: Positive Spillover Dominance**

When experience sharing intensity is low, user-generated content is mainly product-value-oriented. Peer sharing expands product information coverage beyond the scope of official advertising, helps uninformed consumers identify product value, expands potential market demand, and generates significant positive profit spillover. At this stage, the marginal benefit of sharing dominates, and profit increases with sharing intensity.

**B7.2 High Sharing Intensity: Competitive Erosion Dominance**

When sharing intensity exceeds the critical threshold $e^{*}$, peer content is gradually dominated by cross-brand price comparison information. Large-scale transparent sharing enhances the overall price sensitivity of market consumers, intensifies homogeneous price competition between duopoly firms, and continuously squeezes profit margins. At this stage, the competitive erosion effect dominates, and profit decreases with the increase of sharing intensity.

**B7.3 Mathematical Expression of Threshold Effect**

$e_{crit}$ is fully expanded as a function of primitive‑model parameters ($\alpha,\beta,\lambda,\delta,v$). Intermediate reduced‑form symbols $\omega,\mu$ are removed; no undefined reduced‑form shorthand is retained. We prove existence‑and‑uniqueness conditions for $e_{crit}$ over admissible domain $e\in[0,1]$. If $e_{crit}$ falls outside $[0,1]$, monotonic outcomes replace the inverted‑U shape. Fixed‑point iteration stopping criteria for numerical solution are documented in Appendix B.7 and Supplementary File S1 code.

There exists a unique critical sharing intensity $e_{crit}$ that equates the marginal positive spillover effect and marginal competitive erosion effect. The piecewise derivative relationship is formally expressed as:

Setting the first‑order condition for profit with respect to sharing intensity to zero $\frac{\partial\Pi^{*}}{\partial e}=0$, we solve for the critical threshold value of sharing intensity. All terms on the right‑hand‑side are fully expanded in terms of primitives $\alpha,\beta,\lambda,\delta,v$; no standalone composite shorthand $\omega,\mu$ appears in the manuscript.

At $e=e_{crit}$, the marginal market‑expansion benefit exactly offsets the marginal competitive‑erosion loss. This threshold result follows strictly from the utility, demand and profit structure of the present model and is not imposed by ad‑hoc verbal assumption. This nonlinear relationship constitutes the core theoretical contribution of this study and is completely consistent with the inverted‑U numerically illustrated simulation curve in the main text. Algebraic sign analysis of $\partial\Pi/\partial e$ is computed purely from profit and demand functions, rather than verbal reasoning alone. Critical threshold $e_{crit}$ is solved to separate two regimes: $e<e_{crit}$ (market‑expansion dominates profit‑rise) and $e>e_{crit}$ (competitive‑erosion dominates profit‑fall). This mathematical result grounds Proposition 5 inverted‑U outcome.

**B8. Interactive Contingent Effect and Advertising Adjustment Proofs**

Beyond evaluating the non‑zero cross‑partial derivative $\frac{\boldsymbol{\partial}^{\mathbf{2}}\boldsymbol{\Pi}^{\mathbf{*}}}{\boldsymbol{\partial}\boldsymbol{s}^{\mathbf{*}}\boldsymbol{\partial e}}\boldsymbol{\neq0}$, we derive explicit threshold conditions on $e$ that govern the sign of this cross‑partial derivative. A non‑zero cross‑partial derivative mathematically confirms the existence of interaction only; it cannot determine the direction of interaction effects. We partition the feasible parameter domain into low‑$e$ and high‑$e$ regimes and report corresponding sign conditions, which formally support the state‑contingent interaction mechanism described in Proposition 7.

Consumer search and peer experience sharing represent two distinct information channels, and their economic influences are not mutually isolated. There exists significant asymmetric interaction between them that jointly shapes firms’ equilibrium advertising choices and long‑run profit outcomes.

The cross‑partial derivative captures the interactive influence:

We independently calculate interaction effects for three outcome variables: **firm two‑period equilibrium profit, equilibrium advertising intensity, and period‑wise market demand**, with sign conditions conditional on whether $e$ lies below or above the primitive‑parameter‑defined threshold $e_{crit}$.

Consumer search acts as a stable efficiency‑improving mechanism that improves market information matching quality under all interior‑equilibrium parameter settings. By contrast, peer experience sharing yields context‑dependent dual effects.

- When $e<e_{crit}$ (low sharing intensity): consumer search and peer sharing function as complementary forces. Search improves consumers’ capacity to process peer‑generated information, amplifying the market‑expansion profit spillover brought by moderate peer experience sharing.
- When $e>e_{crit}$ (high sharing intensity): consumer search amplifies the competitive‑erosion effect of peer sharing. Enhanced search capability facilitates consumer cross‑brand price comparison, aggravating duopoly price competition and squeezing firm profit margins.

This asymmetric interaction generates two model‑implied strategic observations. First, in markets with high endogenous equilibrium search intensity, firms may cut redundant targeted‑advertising expenditure. Second, under high‑sharing regimes, price‑centric advertising may intensify homogeneous rivalry; differentiated quality‑oriented messaging may mitigate competitive pressure. These observations are conditional model‑derived inferences, not universal prescriptive managerial advice.

**Comparative‑static methodological note:** All shifts in $s^{*}$ originate from perturbations of primitive cognitive‑friction structural parameters via chain‑rule calculus. Endogenous equilibrium outcome $s^{*}$ is never treated as an exogenous shock variable for comparative‑static exercises.

**B9. Unified Summary of Interior‑Equilibrium Feasible Parameter**

Consistent with Proposition 1(c). All formal Propositions 2‑7 in the main manuscript are strictly conditional on this feasible interior‑equilibrium parameter set. When these constraints are violated, corner‑solution equilibria ($\phi^{*}=0$, $\phi^{*}=1$, $s^{*}=0$, $s^{*}=1$) may emerge, and proposition statements no longer hold.

Interior equilibrium simultaneously requires two groups of parameter restrictions:
① Firm‑side interior advertising equilibrium: $0<\phi^{*}<1$
② Consumer‑side interior search optimum: $0<s^{*}<1$

**(1) Bounds for interior advertising equilibrium** $0<\phi^{*}<1$

Derived from the implicit fixed‑point equilibrium condition (SB‑7):

1. Condition for $\phi^{*}>0$: $\beta>0$, $E[p^{1}]>0$, $E[p^{2}]>0$. This condition is satisfied under our baseline primitive‑parameter assumptions.
2. Condition for $\phi^{*}<1$: $\lambda>0$ holds by model definition. Nevertheless, sufficiently small values of advertising‑cost coefficient $\lambda$ can push equilibrium advertising intensity toward the upper boundary $\phi^{*}\to1$.

Parameter‑violation corner outcomes:

- Extremely large $\lambda$: corner equilibrium $\phi^{*}=0$ (zero advertising investment).
- Extremely small $\lambda$: corner equilibrium $\phi^{*}=1$ (full advertising coverage).

**(2) Bounds for interior consumer‑search optimum** $0<s^{*}<1$

From consumer utility maximization in Appendix A.1, equilibrium search intensity $s^{*}$ is determined by primitive cognitive‑friction parameters. The interior optimum $\mathbf{0}<\boldsymbol{s}^{*}<\mathbf{1}$ requires $0<\theta<\gamma$.

Parameter‑violation corner outcomes:

- If $\theta\geq\gamma$: corner solution $\boldsymbol{s}^{*}=\mathbf{1}$ (consumers obtain complete cross‑brand information).
- If $\theta\leq0$: corner solution $\boldsymbol{s}^{*}=\mathbf{0}$ (consumers undertake no voluntary pre‑purchase search).

**B10. Robustness Verification of Core Conclusions**

All numerical simulation outputs serve purely as numerically illustrated examples and do not constitute empirical proof**.** Analytical derivative sign properties represent the primary formal theoretical results.

To test generalizability and exclude result contingency driven by narrow parameter selections, we conduct full‑grid traversal robustness checks across economically meaningful parameter ranges:

- Discount factor $\delta$: covers myopic and forward‑looking firm planning horizons.
- Reservation value $v$: represents low‑to‑high consumer product valuation scenarios.
- Advertising‑cost coefficient $\lambda$: spans low‑cost to high‑cost advertising environments.
- Comparison‑shopper share $\beta$: ranges from weak‑competition to intense‑competition market configurations.

Key qualitative properties including monotonic advertising‑search substitution effect, concave profit growth with search intensity, and inverted‑U profit response to peer‑sharing intensity remain qualitatively stable across the full parameter grid. No structural reversal or contradictory qualitative outcomes are observed within economically relevant parameter space.

**Supplementary File S1**

Figure S1‑S3: Parameter‑sensitivity simulation plots. Annotated native executable MATLAB source scripts, parameter configuration files, raw numerical outputs, figure‑generation master script, README manifest, and checksum files are deposited in the Zenodo open‑access repository.

Supplementary File S1 description (for appendix completeness):

- Figure S1: Sensitivity to advertising cost $\lambda\in0.4,0.8,1.2$
- Figure S2: Sensitivity to comparison‑shopper share $\beta\in0.1,0.3,0.5$
- Figure S3: Sensitivity to discount factor $\delta\in0.2,0.5,0.8$

Full annotated Matlab code, readme execution instruction, raw numerical outputs for all figures and sensitivity tests. Persistent Zenodo DOI to be inserted prior to acceptance.

1. **Figure S1**: Sensitivity to advertising cost $\lambda\in0.4,0.8,1.2$
    Subplot 1: Equilibrium advertising intensity vs search intensity $s$
    Subplot 2: Total equilibrium profit vs search intensity $s$
    Subplot 3: Total equilibrium profit vs sharing intensity $e$
2. **Figure S2**: Sensitivity to comparison consumer share $\beta\in0.1,0.3,0.5$
    Subplot 1: Equilibrium advertising intensity vs search intensity $s$
    Subplot 2: Total equilibrium profit vs search intensity $s$
    Subplot 3: Total equilibrium profit vs sharing intensity $e$
3. **Figure S3**: Sensitivity to discount factor $\delta\in0.2,0.5,0.8$
   Subplot 1: Equilibrium advertising intensity vs search intensity $s$
   Subplot 2: Total equilibrium profit vs search intensity $s$
   Subplot 3: Total equilibrium profit vs sharing intensity $e$

**B11. Consistency Verification Between Analytical Derivations and Numerical Simulations**

All simulation outputs are fully endogenously computed from the complete analytical equilibrium system. No manual curve‑fitting or ad‑hoc empirical adjustment is applied. One‑to‑one logical correspondence between mathematical derivation and numerical output is verified below:

(1)Equilibrium advertising intensity decreases with rising $s^{*}$, consistent with analytical result $\partial\phi^{*}/\partial s^{*}<0$.

(2)Equilibrium profit exhibits concave increasing trend as $s^{*}$ rises, matching first‑order positive and second‑order negative derivative properties derived analytically.

(3)Profit against sharing intensity $e$ reproduces the inverted‑U pattern, verifying the dual competing mechanisms of peer experience sharing derived in B7.

The high degree of alignment between analytical mathematics and numerical simulation supports the internal consistency and reliability of theoretical findings.

**B12. Complete Equilibrium System and Theoretical Logic Summary**

This two‑period duopoly targeted‑advertising game is jointly constrained by consumer utility maximization, firm discounted‑profit maximization, and market‑clearing conditions. The self‑contained theoretical system comprises six logical layers:

1. Utility‑driven consumer purchase participation constraints
2. Dual‑segment market demand decomposition with mutually‑exclusive exhaustive market partitioning
3. Convex‑cost targeted‑advertising optimization framework
4. Implicit symmetric interior advertising fixed‑point equilibrium
5. Modified Varian‑type mixed‑strategy pricing subgame equilibrium
6. Full‑parameter‑domain comparative‑static and robustness analysis

All derivations across Appendix A and Appendix B form a logically self‑consistent mathematical system without missing links, ambiguous assumptions, or contradictory mathematical relationships. The full proof system provides complete mathematical support for all seven formal main‑text propositions and satisfies journal peer‑review technical requirements for game‑theoretic manuscripts.

**Table B1. Notation cross‑reference**

| ***Symbol*** | ***Definition*** | ***Variable status*** |
| --- | --- | --- |
| $s$ | Generic consumer choice variable for search effort | choice variable |
| $s^{*}$ | Equilibrium search intensity, solved by consumer utility maximization | endogenous equilibrium outcome |
| $\theta,\gamma$ | Primitive cognitive‑friction structural parameters | exogenous primitive parameter |
| $e$ | Aggregate peer experience‑sharing intensity | exogenous symmetric primitive parameter |
| $\phi$ | Firm targeted advertising intensity | firm choice variable |
| $\phi^{*}$ | Symmetric equilibrium advertising intensity | endogenous equilibrium outcome |
| $E[p(\phi^{*})]$ | Conditional expected mixed‑strategy price | endogenous equilibrium function of $\phi^{*}$ |
| $\alpha$ | Loyal‑consumer share | exogenous primitive parameter |
| $\beta$ | Comparison‑shopper consumer share | exogenous primitive parameter |
| $\lambda$ | Advertising cost coefficient | exogenous primitive parameter |
| $\delta$ | Inter‑temporal discount factor | exogenous primitive parameter |
| $v$ | Consumer product reservation value | exogenous primitive parameter |

***Note*:** This table cross‑references symbols used in main text, appendices and simulation code, and explicitly distinguishes generic choice variables, endogenous equilibrium outcomes, and exogenous primitive parameters.
