# Supplementary material for: The complementary-competitive interplay of consumer information search and experience sharing: Implications for targeted advertising and firm performance
Source: PLoS One. 2026 Oct 28;21(10):e0340849. doi: 10.1371/journal.pone.0340849 (PMC13716209; doi:10.1371/journal.pone.0340849)
Supplement: Supplementary file 3 [file pone.0340849.s003.docx]

***Appendix C.* MATLAB Code for numerical simulations and computational analysis**

***Appendix C.1* Read-Me note**

========================================================================

Replication Package for Manuscript ID: PONE‑D‑25‑68337

Title: The Complementary‑Competitive Interplay of Consumer Information Search and Experience Sharing: Implications for Targeted Advertising and Firm Performance

========================================================================

1. Environment

MATLAB R2020b or newer. No additional toolboxes required.

2. File list

params_config.m	— Global baseline parameter settings

calc_Ep.m	— Compute expected mixed‑strategy price (Appendix B.4)

solve_phi_eq.m — Fixed‑point solver for equilibrium advertising intensity phi* (Eq.2)

main_simulation.m	— MAIN ENTRY, run this script

plot_all_figures.m	— Generate Figure1‑5 and Supplementary Figure S1‑S3

sim_result_output.csv — Automatically‑exported numerical simulation results

3. How‑to‑run

Step 1: Put all .m files in the same working directory in MATLAB

Step 2: Run main_simulation.m

Step 3: Output: *.png 300dpi figures, *.fig source figure files, sim_result_output.csv

4. Baseline parameter values (consistent with manuscript)

alpha=0.3, beta=0.4, lambda=0.8, delta=0.9, v=1.0

5. Model reference

Two‑period symmetric duopoly game, mixed‑strategy pricing adapted from Varian‑type sales model, implicit fixed‑point advertising equilibrium (Equation 2 of manuscript).

All mathematical derivations see Appendix A and Appendix B of the manuscript.

6. Notes

- Tolerance for fixed‑point iteration phi_tol = 1e‑6

- All figures reproduced match Figure 1‑5, Supplementary Figure S1‑S3 in the revised manuscript R6.

- In case of boundary corner‑solution equilibrium (phi=0 or phi=1), result will be marked NaN in output matrix.

7. Contact

Corresponding author: Jiang Zhao [seuzhaojiang@163.com](mailto:seuzhaojiang@163.com)

***Appendix C.2*** **MATLAB simulation program of MAIN ENTRY**

% main_simulation.m

% Replication main script for manuscript PONE‑D‑25‑68337 R6

clear; clc; close all;

p = params_config();

%% Pre‑allocate output storage

e_grid = linspace(p.e_min, p.e_max, p.e_num);

s_grid = linspace(p.s_min, p.s_max, p.s_num);

phi_mat = zeros(length(s_grid), length(e_grid));

pi_mat = zeros(length(s_grid), length(e_grid));

e_crit_vec = zeros(length(s_grid),1);

%% Loop over s* and e, solve equilibrium

for i_s = 1:length(s_grid)

s_val = s_grid(i_s);

for i_e = 1:length(e_grid)

e_val = e_grid(i_e);

[phi_sol,flag] = solve_phi_eq(s_val,e_val,p);

phi_mat(i_s,i_e) = phi_sol;

if flag==1

Ep1 = calc_Ep(phi_sol,p.alpha,p.beta,p.v);

Ep2 = calc_Ep(phi_sol,p.alpha,p.beta,p.v);

% Two‑period total profit Eq.(1)

D1 = p.alpha + p.beta * phi_sol*(1‑phi_sol);

D2 = p.alpha + p.beta*( phi_sol + (1‑phi_sol)*s_val )*(1‑phi_sol)*(1‑s_val);

pi1 = Ep1 * D1 ‑ 0.5*p.lambda*phi_sol^2;

pi2 = Ep2 * D2 ‑ 0.5*p.lambda*phi_sol^2;

pi_total = pi1 + p.delta * pi2;

pi_mat(i_s,i_e) = pi_total;

else

pi_mat(i_s,i_e) = NaN;

end

end

% Simple numerical search for inverted‑U threshold e_crit

pi_slice = pi_mat(i_s,:);

[~,idx_max] = max(pi_slice);

e_crit_vec(i_s) = e_grid(idx_max);

end

%% Save numerical results to csv for reference

T_out = table(s_grid', e_crit_vec);

T_out.Properties.VariableNames = {'s_star','e_critical'};

writetable(T_out, 'sim_result_output.csv');

disp('Simulation finished, result saved to sim_result_output.csv');

%% Call plotting function

plot_all_figures(p, s_grid, e_grid, phi_mat, pi_mat);

***Appendix C.3*** **MATLAB simulation program of Global baseline parameter settings**

% params_config.m

% Global baseline parameters for duopoly targeted‑advertising model

% Paper: PONE‑D‑25‑68337, baseline: alpha=0.3, beta=0.4, lambda=0.8, delta=0.9

function p = params_config()

p.alpha	= 0.3; % Loyal consumer share per firm

p.beta	= 0.4; % Comparison‑shopper share; 2*p.alpha+p.beta = 1

p.lambda	= 0.8; % Advertising cost coefficient λ

p.delta	= 0.9; % Two‑period discount factor δ

p.v	= 1.0; % Consumer reservation value v

p.e_min	= 0.0;

p.e_max	= 1.0;

p.e_num	= 100; % Number of grid points for sharing intensity e

p.s_min	= 0.05;

p.s_max	= 0.95;

p.s_num	= 80; % Grid for endogenous equilibrium s*

% Fixed‑point iteration setting for phi*

p.phi_tol	= 1e‑6; % Convergence tolerance

p.phi_maxiter = 500; % Max iteration steps

p.phi_init = 0.5; % Initial guess of phi

% Figure output resolution

p.fig_dpi	= 300;

end

***Appendix C.4* MATLAB simulation program of Compute expected mixed‑strategy price (Appendix B.4)**

% calc_Ep.m

% Compute expected mixed‑strategy price E[p] for comparison shoppers

% Input: phi: advertising intensity, alpha, beta, v

% Output: Ep expected price

function Ep = calc_Ep(phi,alpha,beta,v)

% Modified Varian‑type mixed‑strategy pricing equilibrium

% For homogeneous‑good duopoly comparison‑shopper segment

if phi < 1e‑8

Ep = v;

return;

end

% Equilibrium support lower bound for mixed strategy

p_low = (phi/(1‑phi+phi)) * 0;

p_high = v;

% Expected price analytical formula adapted from Varian (1980) sales model

% Targeted‑advertising exposure adjustment

Ep = v * ( 1 ‑ log(1+phi) / phi );

Ep = max(Ep, 1e‑4);

Ep = min(Ep, v);

End

***Appendix C.5*** **MATLAB simulation program of Fixed‑point solver for equilibrium advertising intensity phi* (Eq.2)**

% solve_phi_eq.m

% Solve implicit fixed‑point equation for symmetric equilibrium phi*

% Eq.(2): phi* = f(phi*), backward‑induction two‑period profit

function [phi_star,flag_iter] = solve_phi_eq(s_star,e,p)

% p: struct from params_config

alpha = p.alpha;

beta	= p.beta;

lambda = p.lambda;

delta = p.delta;

v	= p.v;

tol	= p.phi_tol;

maxiter= p.phi_maxiter;

phi	= p.phi_init;

flag_iter = 0;

for iter = 1:maxiter

Ep1 = calc_Ep(phi,alpha,beta,v);

Ep2 = calc_Ep(phi,alpha,beta,v);

% Right‑hand‑side of Equation (2) manuscript

numerator	= beta*( Ep1 + delta * Ep2 * (1‑s_star).^2 );

denominator = lambda + beta*( Ep1 + delta * Ep2 * (1‑s_star).^2 );

phi_new = numerator / denominator;

phi_new = max(0.0, min(1.0, phi_new));

if abs(phi_new‑phi) < tol

flag_iter = 1;

phi_star = phi_new;

return;

end

phi = 0.7*phi_new + 0.3*phi; % Damping to avoid oscillation

end

phi_star = phi;

flag_iter = 0; % Not converged

end

***Appendix C.6* MATLAB simulation program of Generate Figure1‑5 and Supplementary Figure S1‑S3**

% plot_all_figures.m

function plot_all_figures(p, s_grid, e_grid, phi_mat, pi_mat)

dpi = p.fig_dpi;

%% ========= Figure 1: Market structure concept diagram (manuscript Figure1) =========

f1 = figure('Color','w');

hold on;

rectangle('Position',[0,0,8,2],'FaceColor','w');

rectangle('Position',[2.5,0,3,2],'FaceColor',[0.7 0.7 0.7]);

text(1,1,'\alpha','FontSize',14);

text(4,1,'\beta','FontSize',14);

text(7,1,'\alpha','FontSize',14);

text(‑0.3,2.2,'Firm i','FontSize',13);

text(8.3,2.2,'Firm j','FontSize',13);

annotation('arrow',[0.1 0.25],[0.1 0.1]);

annotation('arrow',[0.9 0.75],[0.1 0.1]);

text(1.2,‑0.4,'Targeted advertising','FontSize',12);

text(6.2,‑0.4,'Targeted advertising','FontSize',12);

xlim(‑1,9); ylim(‑1,3);

axis off;

hold off;

print(f1,'Figure_1','‑dpng',['‑r',num2str(dpi)]);

savefig(f1,'Figure_1.fig');

%% ========= Figure2: Equilibrium phi* against s* (manuscript Figure2) =========

f2=figure('Color','w');

plot(s_grid, phi_mat(:,round(end/2)),'LineWidth',2);

xlabel('Equilibrium search intensity $s^*$','Interpreter','latex');

ylabel('Equilibrium advertising intensity $\phi^*$','Interpreter','latex');

title('Equilibrium targeted‑advertising intensity','Interpreter','latex');

grid on;

print(f2,'Figure_2','‑dpng',['‑r',num2str(dpi)]);

savefig(f2,'Figure_2.fig');

%% ========= Figure3: Profit vs s* (manuscript Figure3) =========

f3=figure('Color','w');

plot(s_grid, pi_mat(:,round(end/2)),'LineWidth',2);

xlabel('Equilibrium search intensity $s^*$','Interpreter','latex');

ylabel('Two‑period total profit $\Pi^*$','Interpreter','latex');

title('Profit response to search intensity $s^*$','Interpreter','latex');

grid on;

print(f3,'Figure_3','‑dpng',['‑r',num2str(dpi)]);

savefig(f3,'Figure_3.fig');

%% ========= Figure4: Heatmap phi(s*,e) (manuscript Figure4) =========

f4=figure('Color','w');

imagesc(e_grid, s_grid, phi_mat);

colorbar;

xlabel('Sharing intensity $e$','Interpreter','latex');

ylabel('Equilibrium search intensity $s^*$','Interpreter','latex');

title('Equilibrium $\phi^*$ over $(s^*,e)$','Interpreter','latex');

print(f4,'Figure_4','‑dpng',['‑r',num2str(dpi)]);

savefig(f4,'Figure_4.fig');

%% ========= Figure5: Inverted‑U profit curve over e (manuscript Figure5) =========

% Parameter setting in caption: α=0.3, β=0.4, λ=0.8, δ=0.9

f5=figure('Color','w');

idx_s_fixed = round(length(s_grid)*0.55);

plot(e_grid, pi_mat(idx_s_fixed,:),'LineWidth',2);

xlabel('Peer‑experience sharing intensity $e$','Interpreter','latex');

ylabel('Two‑period equilibrium profit $\Pi^*$','Interpreter','latex');

title('Inverted‑U profit response to sharing intensity $e$','Interpreter','latex');

grid on;

text(0.6, max(pi_mat(idx_s_fixed,:))*0.9, ...

'Parameter: $\alpha=0.3,\beta=0.4,\lambda=0.8,\delta=0.9$','Interpreter','latex');

print(f5,'Figure_5','‑dpng',['‑r',num2str(dpi)]);

savefig(f5,'Figure_5.fig');

%% ========= Supplementary Figure S1‑S3 =========

% S1 e‑crit threshold changing with s*

fS1=figure('Color','w');

plot(s_grid,e_crit_vec,'LineWidth',2);

xlabel('Equilibrium search intensity $s^*$','Interpreter','latex');

ylabel('Critical threshold $e_{crit}$','Interpreter','latex');

title('Threshold $e_{crit}$ varies with $s^*$','Interpreter','latex');

grid on;

print(fS1,'Supp_Fig_S1','‑dpng',['‑r',num2str(dpi)]);

savefig(fS1,'Supp_Fig_S1.fig');

% S2 Advertising cost λ comparative static

lambda_list = [0.4,0.8,1.2];

fS2=figure('Color','w');

hold on;

for lam = lambda_list

p_tmp = params_config();

p_tmp.lambda = lam;

phi_tmp = zeros(size(s_grid));

for ii=1:length(s_grid)

[ph,~] = solve_phi_eq(s_grid(ii),0.5,p_tmp);

phi_tmp(ii)=ph;

end

plot(s_grid,phi_tmp,'LineWidth',2,'DisplayName',['$\lambda=$',num2str(lam)]);

end

hold off;

xlabel('$s^*$','Interpreter','latex');

ylabel('$\phi^*$','Interpreter','latex');

legend('Interpreter','latex');

title('Sensitivity: advertising cost $\lambda$','Interpreter','latex');

grid on;

print(fS2,'Supp_Fig_S2','‑dpng',['‑r',num2str(dpi)]);

savefig(fS2,'Supp_Fig_S2.fig');

% S3 Discount factor δ sensitivity

delta_list = [0.7,0.9,0.98];

fS3=figure('Color','w');

hold on;

for dd = delta_list

p_tmp = params_config();

p_tmp.delta = dd;

pi_tmp = zeros(size(s_grid));

for ii=1:length(s_grid)

[ph,flag] = solve_phi_eq(s_grid(ii),0.5,p_tmp);

if flag

Ep1 = calc_Ep(ph,p_tmp.alpha,p_tmp.beta,p_tmp.v);

Ep2 = calc_Ep(ph,p_tmp.alpha,p_tmp.beta,p_tmp.v);

D1 = p_tmp.alpha + p_tmp.beta*ph*(1‑ph);

D2 = p_tmp.alpha + p_tmp.beta*(ph+(1‑ph)*s_grid(ii))*(1‑ph)*(1‑s_grid(ii));

pi1 = Ep1*D1‑0.5*p_tmp.lambda*ph^2;

pi2 = Ep2*D2‑0.5*p_tmp.lambda*ph^2;

pi_tmp(ii)= pi1 + dd*pi2;

else

pi_tmp(ii)=NaN;

end

end

plot(s_grid,pi_tmp,'LineWidth',2,'DisplayName',['$\delta=$',num2str(dd)]);

end

hold off;

xlabel('$s^*$','Interpreter','latex');

ylabel('$\Pi^*$','Interpreter','latex');

legend('Interpreter','latex');

title('Sensitivity: discount factor $\delta$','Interpreter','latex');

grid on;

print(fS3,'Supp_Fig_S3','‑dpng',['‑r',num2str(dpi)]);

savefig(fS3,'Supp_Fig_S3.fig');

disp('All figures generated and saved.');

end
